# HPLC Separation of Diastereomers: Chiral Molecular Tools Useful for the Preparation of Enantiopure Compounds and Simultaneous Determination of Their Absolute Configurations

**DOI:** 10.3390/molecules21101328

**Published:** 2016-10-04

**Authors:** Nobuyuki Harada

**Affiliations:** Institute of Multidisciplinary Research for Advanced Materials, Tohoku University, 2-1-1 Katahira, Aoba, Sendai 980-8577, Japan; n2harada@tagen.tohoku.ac.jp

**Keywords:** chiral molecule, absolute configuration, diastereomers, HPLC separation on silica gel, ^1^H-NMR diamagnetic anisotropy, X-ray crystallography, internal reference of absolute configuration

## Abstract

To obtain enantiopure compounds, the so-called chiral high performance liquid chromatography (HPLC) method, i.e., HPLC using a chiral stationary phase, is very useful, as reviewed in the present Special Issue. On the other hand, normal HPLC (on silica gel) separation of diastereomers is also useful for the preparation of enantiopure compounds and also for the simultaneous determination of their absolute configurations (ACs). The author and coworkers have developed some chiral molecular tools, e.g., camphorsultam dichlorophthalic acid (CSDP acid), 2-methoxy-2-(1-naphthyl)propionic acid (MαNP acid), and others suitable for this purpose. For example, a racemic alcohol is esterified with (*S*)-(+)-MαNP acid, yielding diastereomeric esters, which are easily separable by HPLC on silica gel. The ACs of the obtained enantiopure MαNP esters can be determined by the ^1^H-NMR diamagnetic anisotropy method. In addition, MαNP or CSDP esters have a high probability of giving single crystals suitable for X-ray crystallography. From the X-ray Oak Ridge thermal ellipsoid plot (ORTEP) drawing, the AC of the alcohol part can be unambiguously determined because the AC of the acid part is already known. The hydrolysis of MαNP or CSDP esters yields enantiopure alcohols with the established ACs. The mechanism and application examples of these methods are explained.

## 1. Introduction

**Figure 1:**
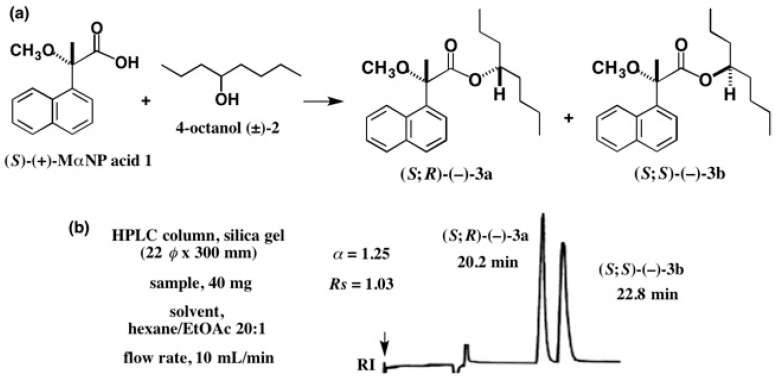
Preparation of diastereomeric 4-octanol (*S*)-(+)-2-methoxy-2-(1-naphthyl)propionic acid (MαNP) esters **3a** and **3b** (**a**) and separation by high performance liquid chromatography (HPLC) on silica gel (**b**). Redrawn with permission from [13].

It is well known that the so-called chiral high performance liquid chromatography (HPLC) method, i.e., HPLC with a chiral stationary phase, is very useful for the separation of enantiomers, i.e., the preparation of enantiopure compounds [1,2,3,4,5], as will be explained in other review articles and/or research papers reported in this Special Issue. On the other hand, there is another HPLC method for the preparation of enantiopure compounds, where diastereomers are separated by normal HPLC on silica gel [6,7,8,9,10,11], or by reversed-phase HPLC [12]. For example, racemic 4-octanol (±)-**2** was esterified with (*S*)-(+)-2-methoxy-2-(1-naphthyl)propionic acid **1** (MαNP acid) yielding a diastereomeric mixture of esters **3a** and **3b** (Figure 1) [13]. It was surprising to find that diastereomeric esters **3a** and **3b** (40 mg sample) could be completely separated by HPLC on silica gel (separation factor α = 1.25; resolution factor *Rs* = 1.03), regardless of the very small chirality of the 4-octanol moiety, which is generated by the difference between propyl (C_3_) and butyl (C_4_) groups; especially since both are normal chain alkyl groups. Please note that in this review article, the diastereomers are designated by compound numbers., e.g., **3a** and **3b**, where small letter **a** indicates the first-eluted fraction in HPLC, and **b** is the second-eluted one.

**Figure 2:**
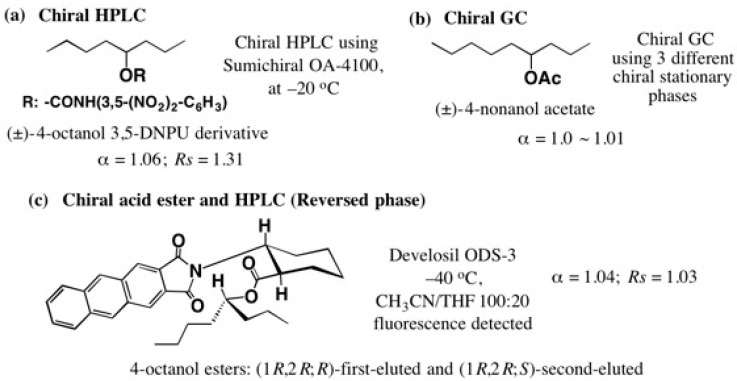
HPLC or gas chromatography (GC) separation of alkyl chain alcohol derivatives (**a**)–(**c**).

In general, it is very difficult to separate enantiomers or diastereomers composed of C, H, and O atoms by HPLC or gas chromatography (GC), especially in the case of aliphatic chain compounds. For example, Figure 2 shows the separation results of 4-octanol or 4-nonanol, reported to date: (a) a racemic 4-octanol 3,5-DNPU (3,5-dinitrophenylurethane) derivative was subjected to chiral HPLC at −20 °C, but the separation factor α remained low as α = 1.06 [14]; (b) separation of racemic 4-nonanol acetate was attempted by chiral GC, but this was not successful, α = 1.0~1.01 [15]; (c) racemic 4-octanol was esterified with a chiral acid yielding diastereomers, which were separated by HPLC (reversed phase) at −40 °C, but α remained low as α = 1.04 [16].

In contrast, as shown in Figure 1, MαNP esters **3a** and **3b** were more effectively separated, indicating that MαNP acid is especially useful for such separation. Therefore, our first purpose is to develop chiral molecular tools such as MαNP acid for effective diastereomers separation by HPLC on silica gel, and then to obtain enantiopure compounds from the separated diastereomers, as will be explained in this review article.

There is another resolution method where diastereomeric ionic crystals, e.g., acid/amine, or inclusion complex crystals are fractionally recrystallized to separate diastereomers. If good crystals are obtained, the method is useful for separation on a large scale. However, the separated crystals are not always diastereomerically pure, despite many crystallizations. If so, it is difficult to obtain enantiopure target compounds. This is the reason why we have not used the ionic crystals or inclusion crystals, but selected covalently bonded diastereomers such as esters or amides, to which HPLC on silica gel is applicable for separation and further purification.

Our second purpose is to determine the absolute configuration (AC). It is well known that the X-ray Bijvoet method [17] using the heavy atom effect, the circular dichroism (CD) exciton chirality method [18], and the recent density functional theory (DFT) molecular orbital calculation [19] are all very useful as non-empirical methods of AC determination. There is another category of AC determination where the relative configuration against the internal reference of AC could be determined by X-ray crystallography and/or ^1^H-NMR diamagnetic anisotropy methods [6,7,8,9,10,11]. If single crystals suitable for X-ray diffraction are available and the final Oak Ridge thermal ellipsoid plot (ORTEP) drawing is obtained, it is very easy to determine the AC of the part in question, based on the AC of the internal reference. This X-ray internal reference method is the most straightforward and reliable.

**Figure 3:**
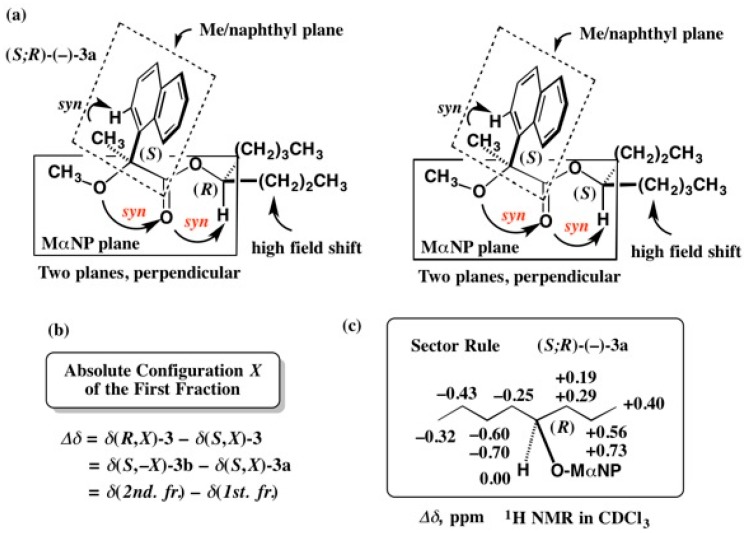
Determination of absolute configurations of 4-octanol MαNP esters (−)-**3a** and (−)-**3b** by ^1^H-NMR diamagnetic anisotropy: (**a**) Preferred conformations; (**b**) Definition of *Δδ* value; (**c**) Sector rule. Redrawn with permission from [13].

Another relative method is the ^1^H-NMR diamagnetic anisotropy method, that is explained in Figure 3 [13]. The first-eluted MαNP ester (−)-**3a** and the second-eluted ester (−)-**3b** take the preferred conformations as shown in Figure 3a. In ester (−)-**3a**, the propyl group is located above the naphthyl group plane, and hence the protons of the propyl group feel the diamagnetic anisotropy effect leading to a high-field shift. On the other hand, in ester (−)-**3b**, the butyl group protons are placed above the naphthalene ring, leading to a high-field shift. The diamagnetic anisotropy effect (∆*δ*) is defined as shown in Figure 3b, where *X* is the AC of the first-eluted ester (−)-**3a** to be determined. The ∆*δ* values were calculated from the observed ^1^H-NMR spectra as shown in Figure 3c, where the propyl group showing positive ∆*δ* values is placed on the right side, while the butyl group giving negative ∆*δ* values is placed on the left side. By applying the present sector rule, the AC of the first-eluted MαNP ester (−)-**3a** was determined to be (*R*) [13].

**Figure 4:**
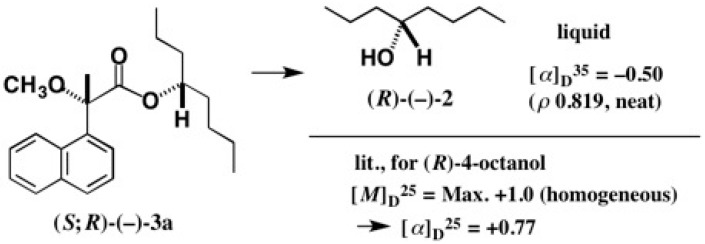
Recovery of enantiopure 4-octanol (*R*)-(−)-**2** and its optical rotation data. The literature data: ${{\left[M\right]}_{D}}^{25}$ value from [20]; ${{\left[\alpha \right]}_{D}}^{25}$ value estimated from ${{\left[M\right]}_{D}}^{25}$ by us.

The first-eluted ester (−)-**3a** was hydrolyzed with KOH/MeOH yielding enantiopure 4-octanol (*R*)-(−)-**2** (yield 70%), whose specific rotation value was negative: ${{\left[\alpha \right]}_{D}}^{35}$ = −0.50 (*ρ* = 0.819, neat) (Figure 4) [13]. To compare with the reported data, we checked the literature, and found only one paper, reported in 1936, where (*R*)-AC was assigned to 4-octanol (+)-**2** (Figure 4) [20]. Namely, the AC assignment was opposite to ours. It was a time before the discovery of the unambiguous AC determination by the X-ray Bijvoet method [17] using the anomalous scattering effect of heavy atoms, and hence the AC assignment in 1936 would be unreliable. Thus, we have first unambiguously determined the (*R*)-AC of 4-octanol (−)-**2**.

We have developed powerful chiral molecular tools suitable for the HPLC separation of diastereomers and also for the determination of ACs. The development and application examples of the methods using these chiral molecular tools will be explained in the following sections.

## 2. Use of (−)-Camphorsultam for Carboxylic Acids

### 2.1. Application to Spiro[3.3]Heptane-Dicarboxylic Acids

**Figure 5:**
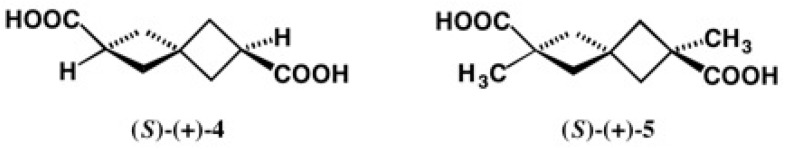
Absolute configurations of Fecht acid (+)-**4** and analog (+)-**5**.

Fecht acid **4** is a unique dicarboxylic acid with spiro[3.3]heptane skeleton (Figure 5). However, it has protons at the α-positions of carboxylic acid groups, and hence it may be unstable under basic conditions. To prevent such a possibility, we designed a Fecht acid analog, 2,6-dimethyl-spiro[3.3]heptane-2,6-dicarboxylic acid (**5**) in which the α-positions are blocked by methyl groups (Figure 5) [21].

**Figure 6:**
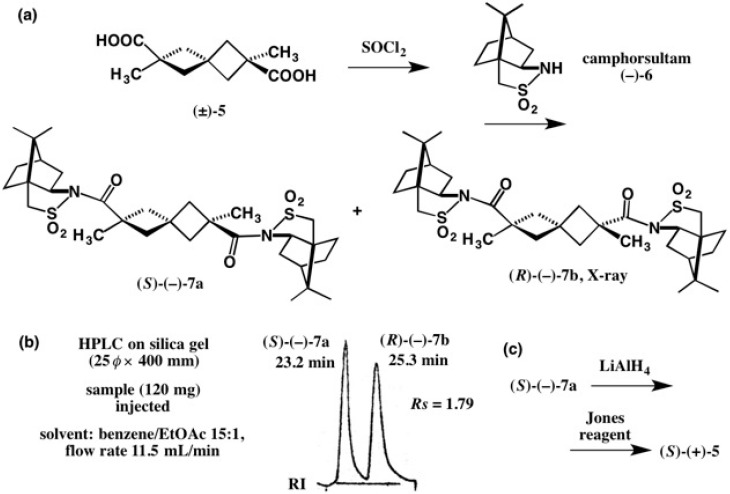
Preparation of diastereomeric amides **7a** and **7b** (**a**), separation by HPLC on silica gel (**b**), and recovery of acid (*S*)-(+)-**5** (**c**). HPLC, redrawn from [22].

To make the chiral resolution of acid **5**, we have tried the HPLC separation of diastereomeric amides formed from chiral amines, such as (*S*)-(−)-α-phenylethylamine, (*S*)-(−)-α-naphthylethyl-amine, and (+)-dehydroabietylamine, but all attempts were unsuccessful. Finally, we have found that (−)-camphorsultam **6** was useful as shown in Figure 6. Racemic acid (±)-**5** was converted into the acid chloride, which was then treated with camphorsultam (−)-**6**/NaH. The obtained diastereomeric mixture (**7a**/**7b**) was well separated by HPLC on silica gel (resolution factor *Rs* = 1.79) (Figure 6b) [21]. Since the sample (120 mg) was separable in one run, this method is good for the preparation of enantiopure target compounds on a laboratory scale. We have a question why camphorsultam amides **7a** and **7b** are more easily separable by HPLC on silica gel than the other amides described above. The author considers that the polar SO_2_ moiety strongly interacts with silica gel.

**Figure 7:**
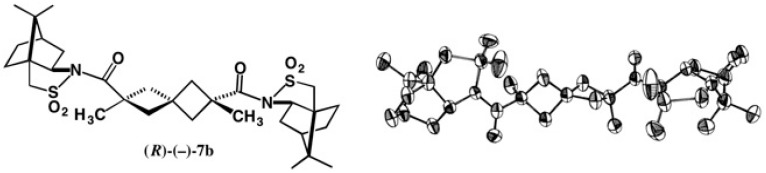
X-ray ORTEP drawing of amide (*R*)-(−)-**7b**. Reprinted from [21].

The second-eluted amide (−)-**7b** was recrystallized from EtOAc giving prisms suitable for X-ray analysis. The diffraction measurements were performed with Cu Kα X-ray giving the ORTEP drawing as shown in Figure 7.

The X-ray final *R* value (resudual factor) was 0.0581 for the (*R*)-AC shown, while that of the mirror image was 0.0650. Therefore, the (*R*)-AC was assigned to amide (−)-**7b** by the X-ray heavy atom effect of the sulfur atom contained in the camphorsultam unit. In addition, the (*R*)-AC of the 2,6-dimethyl-spiro[3.3]heptane-2,6-dicarboxylic acid unit was confirmed by using the camphor- sultam groups as the internal reference of AC. Thus, the AC of the dicarboxylic acid moiety in amide (−)-**7b** was doubly determined by X-ray crystallography; this is a great advantage of the camphorsultam method [21].

To cleave the amide C–N bond, the first-eluted amide (*S*)-(−)-**7a** was reduced with LiAlH4 yielding a bis(primary alcohol), which was then oxidized with the Jones reagent, affording dicarboxylic acid (*S*)-(+)-**5** (Figure 5 and Figure 6c). The camphorsultam method is thus very useful for the preparation of enantiopure carboxylic acids and also for determining their ACs [21].

**Figure 8:**
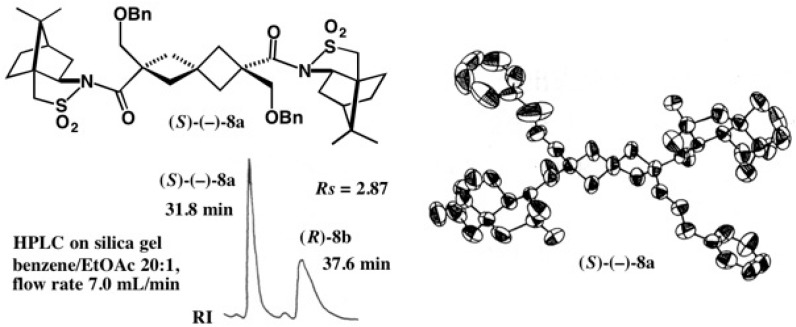
HPLC separation of camphorsultam amides **8a** and **8b**, and an X-ray ORTEP drawing of the first-eluted amide (*S*)-(−)-**8a**. HPLC, redrawn from [24]. X-ray ORTEP drawing, reprinted with permission from [23].

The related diastereomers **8a** and **8b** with benzyloxy groups were largely separated by HPLC on silica gel (Figure 8). It should be emphasized that these bis-amides appeared as clearly separated two spots even on a 5 cm thin layer chromatography (TLC) plate of silica gel. The first-eluted amide (−)-**8a** was recrystallized from EtOAc giving prisms, one of which was subjected to X-ray analysis [23]. From the ORTEP drawing, its (*S*) absolute configuration was established as shown. Amide (*S*)-(−)-**8a** was then converted to dicarboxylic acid (*S*)-(+)-**5** (Figure 5).

### 2.2. Application to Cyclophane-Carboxylic Acids

**Figure 9:**
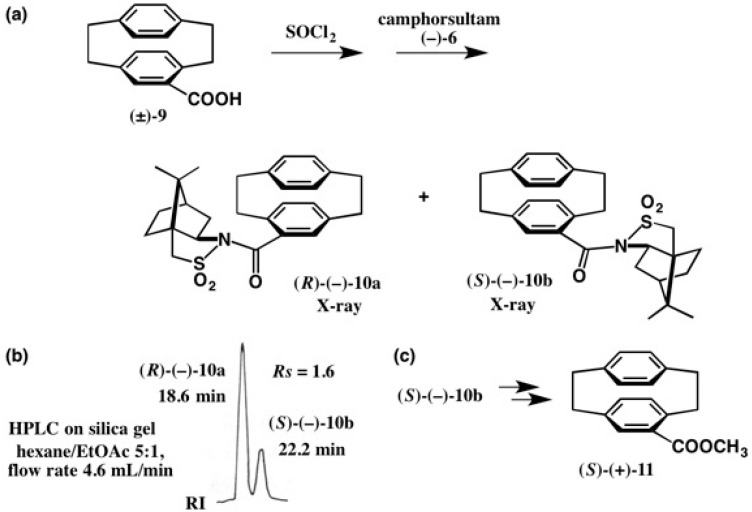
Preparation of diastereomeric amides **10a** and **10b** (**a**), separation by HPLC on silica gel (**b**), and preparation of ester (*S*)-(+)-**11** (**c**). HPLC, redrawn from [24].

The camphorsultam method was useful for the preparation of enantiopure cyclophane compounds with a plane chirality, and also for the direct and clear-cut determination of their ACs. In 1970, Schloegl and coworkers reported the AC of [2.2]paracyclophane-4-carboxylic acid **9** [25]. Acid **9** was enantiomerically enriched by the kinetic resolution of its anhydride with (−)-phenyl-ethylamine, where (*S*)-AC was empirically assigned to acid (+)-**9**. Furthermore, the (*R*)-AC of acid (−)-**9** was also determined by applying the empirical Horeau’s rule to related compounds. However, the AC of acid **9** was later involved in a controversy in relation to the AC determination of other paracyclophane compounds. Therefore, it was necessary to determine its AC in a non-empirical manner (Figure 9) [23].

Racemic acid (±)-**9** was converted to the acid chloride, which was then treated with camphorsultam (−)-**6**/NaH (Figure 9) [23]. The obtained diastereomeric mixture of amides **10a** and **10b** was separated by HPLC on silica gel as shown in Figure 9b. The second-eluted amide (−)-**10b** had a good crystallinity, and so it crystallized before HPLC. Thus, by combining recrystallization and HPLC separation, diastereomeric amides **10a** and **10b** were completely separated.

**Figure 10:**
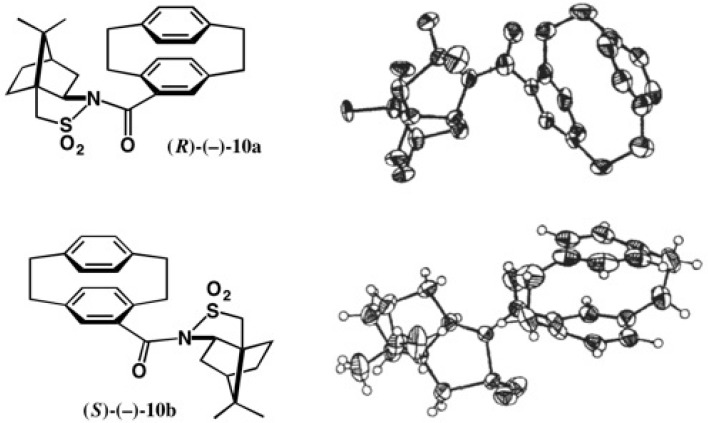
X-ray ORTEP drawings of camphorsultam amides (*R*)-(−)-**10a** and (*S*)-(−)-**10b**. Reprinted with permission from [23].

The second-eluted amide **10b** was recrystallized from EtOAc yielding prisms suitable for X-ray. Based on the AC of the camphorsultam part in the ORTEP drawing, the AC of the cyclophane moiety in (−)-**10b** was unambiguously determined to be (*S*) (Figure 10), which was corroborated by the anomalous scattering effect of the sulfur atom (real image, *R* = 0.0299; mirror image, *R* = 0.0348) [23]. The first-eluted amide (−)-**10a** was recrystallized from MeOH giving prisms; X-ray analysis revealed that one asymmetric unit contained two independent molecules, and hence the final *R* value remained large. Therefore, it was difficult to determine the AC by the heavy atom effect, because of the small difference between final *R* values (*R* = 0.1226 for the real image and *R* = 0.1239 for its mirror image). However, by using the camphorsultam unit as an internal reference of AC, the (*R*)-AC of (−)-**10a** was clearly determined as shown in Figure 10. Finally, amide (*S*)-(−)-**10b** was converted to methyl ester (*S*)-(+)-**11** (Figure 9c). The ACs of [2.2]paracyclophane-4-carboxylic acid **9** and related compounds were thus unambiguously determined [23].

**Figure 11:**
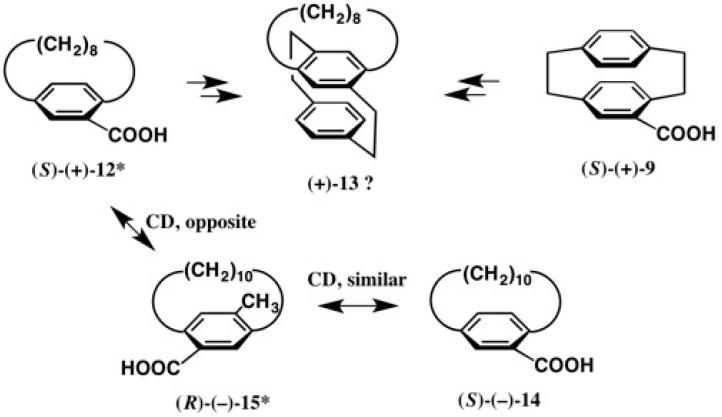
Absolute configurations (ACs) of chiral paracyclophane-carboxylic acids assigned by chemical correlation and comparison of circular dichroism (CD) spectra, where the ACs designated with an asterisk * were reversed, as will be explained below. The AC of compound (+)-**13** has remained unclear. Redrawn with permission from [30].

There had been conflicting reports concerning the ACs of [8]paracyclophane-10-carboxylic acid **12**, [10]paracyclophane-12-carboxylic acid **14**, and a related compound **15** (Figure 11). In 1972, Schloegl and a coworker determined the AC of [10]paracyclophane-12-carboxylic acid **14** as follows [26]. As in the case of [2.2]paracyclophane-4-carboxylic acid **9**, acid **14** was enantiomerically enriched by the kinetic resolution of its anhydride with (−)-phenylethylamine, where (*S*)-AC was empirically assigned to acid (−)-**14**. Furthermore, the (*S*)-AC of acid (−)-**14** was also determined by applying the empirical Horeau’s rule to related compounds.

On the other hand, in 1974–1977, Nakazaki and coworkers reported the synthesis of a unique compound, (+)-[8]bridged [2.2]paracyclophane **13**, and related compounds starting from [8]para-cyclophane-10-carboxylic acid (+)-**12** (Figure 11) [27]. The ACs of these compounds were determined by chemical correlation with [2.2]paracyclophane-4-carboxylic acid (*S*)-(+)-**9**, where chemical reactions of many steps were performed. So, the (*S*)-AC was assigned to acid (+)-**12**. The CD spectrum of acid (*S*)-(+)-[CD(+)248]-**12** was almost a mirror image of that of 15-methyl-[10]paracyclophane-12-carboxylic acid (*R*)-(−)-[CD(−)245]-**15** [27]. On the other hand, the CD of [10]paracyclophane-12-carboxylic acid (*S*)-(−)-[CD(−)239]-**14** reported by Schloegl was similar including a sign to that of acid (−)-**15** despite their opposite ACs (Figure 11) [26]. Thus, these AC assignments were clearly in conflict with each other [28,29]. To solve these problems, we have applied a more unambiguous method, i.e., the camphorsultam method, as explained below [30].

**Figure 12:**
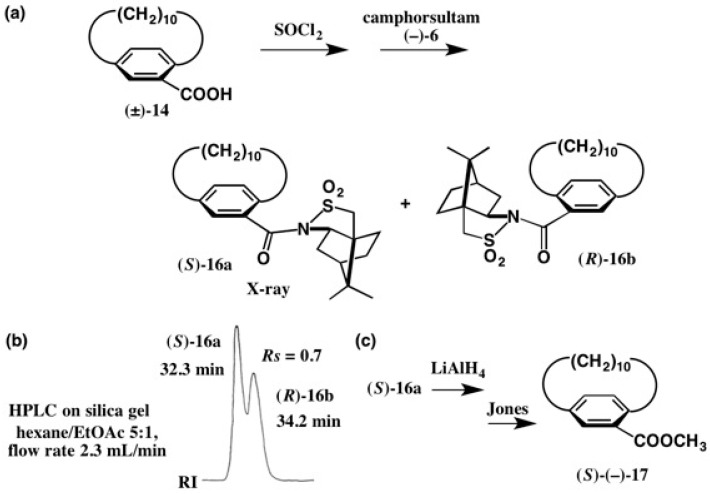
Preparation of diastereomeric amides **16a** and **16b** (**a**), separation by HPLC on silica gel (**b**), and preparation of methyl ester (*S*)-(−)-**17** (**c**). HPLC, redrawn from [24].

Racemic [10]paracyclophane-12-carboxylic acid, (±)-**14** was converted to the acid chloride which was then treated with camphorsultam (−)-**6**/NaH (Figure 12). The obtained diastereomeric mixture of amides **16a** and **16b** was separated by HPLC on silica gel as shown in Figure 12b, where two peaks partially overlap with each other. So, HPLC separation was repeated twice to obtain pure diastereomers [30].

**Figure 13:**
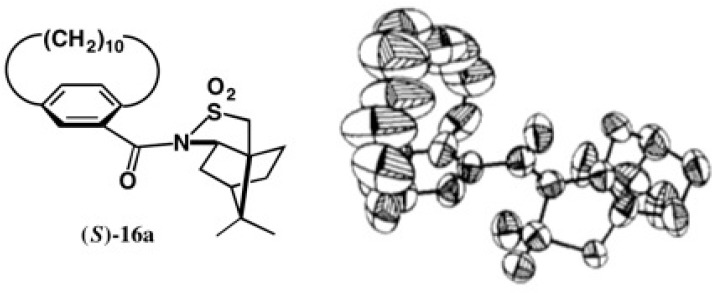
X-ray ORTEP drawing of amide (*S*)-**16a**. Reprinted with permission from [30].

The second-eluted amide **16b** had a good crystallinity, and it was recrystallized from MeOH giving prisms. However, X-ray experiments indicated that the asymmetrical unit contained three independent molecules, and hence it was difficult to continue the X-ray analysis. The first-eluted amide **16a** was similarly recrystallized from MeOH giving plate crystals. Although the asymmetrical unit contained two independent molecules, it was possible to obtain the crystal structure as shown in Figure 13 [30].

As seen in the ORTEP drawing, the methylene chain showed large thermal vibration and/or disorder, and so the final *R* value remained large (*R* = 0.1119 for the real image and *R* = 0.1122 for mirror image). Thus, it was impossible to determine the AC by the anomalous scattering effect of the sulfur atom. However, it was easy to assign the (*S*)-AC of the paracyclophane part from the ORTEP drawing, because the AC of the camphorsultam unit was already known. The first-eluted amide (*S*)-**16a** was converted to methyl ester (*S*)-(−)-[CD(−)238.2]-**17** [30], the CD data of which were similar to those of acid (−)-**14** [26]. This X-ray result was consistent with the (*S*)-AC of acid (−)-**14** previously assigned by the empirical methods [26].

**Figure 14:**
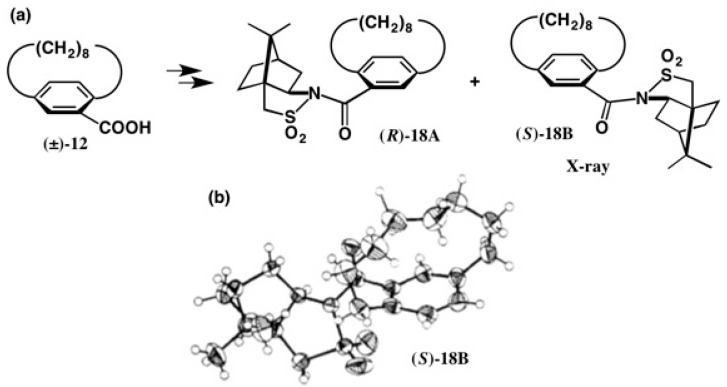
Preparation of amides **18A** and **18B** (**a**), and the X-ray ORTEP drawing of camphorsultam amide (*S*)-**18B** (**b**). Reprinted with permission from [30].

Next, the camphorsultam method was applied to [8]paracyclophane-10-carboxylic acid **12**. Racemic acid (±)-**12** was converted to diastereomeric amides **18A** and **18B** (Figure 14), which were subjected to HPLC, but unfortunately the amides could not be separated. (Please note that in compounds **18A** and **18B**, the capital letters **A** and **B** do not indicate HPLC elution order, just meaning two diastereomers). So, the fractional recrystallization from MeOH was applied, giving amide **18B** as plate single crystals suitable for X-ray crystallography. The ORTEP drawing of amide **18B** was obtained as shown in Figure 14b. The (*S*)-AC of **18B** was determined by the heavy atom effect (*R* = 0.0441 for the real image and *R* = 0.0538 for its mirror image). This assignment was also corroborated by the internal reference method of camphorsultam [30].

**Figure 15:**
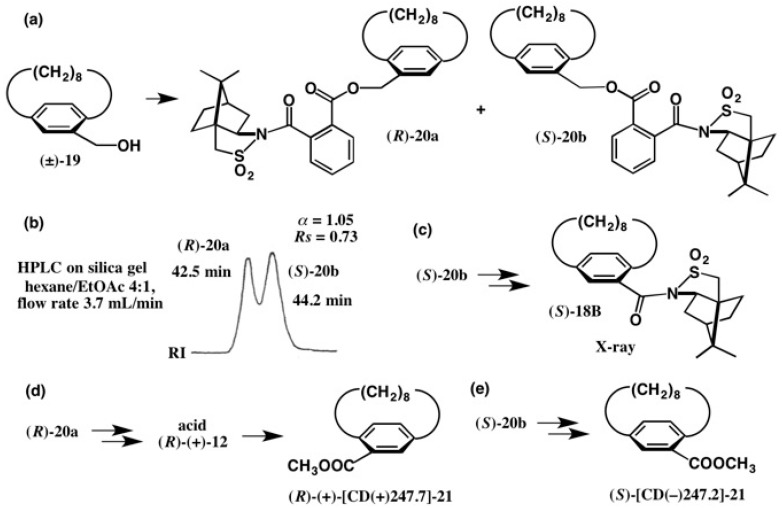
Preparation (**a**) and HPLC separation (**b**) of esters **20a** and **20b**, which were then converted to methyl esters (*R*)-(+)-**21** (**d**) and (*S*)-**21** (**e**), respectively. Ester **20b** was converted to amide **18B** (**c**), by which the ACs of these compounds were established. HPLC, redrawn from [24].

**Figure 16:**
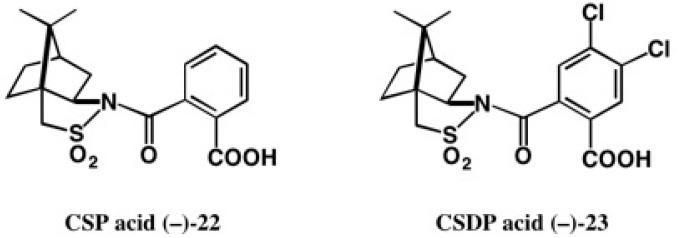
Chiral (−)-CSP and (−)-CSDP acids useful for alcohols.

The amount of amide **18B** obtained by recrystallization was limited, and so we adopted the method of camphorsultam-phthalic acid (CSP acid) (−)-**22** (see Figure 16), which had been developed by us for the chiral resolution of alcohols, as will be discussed in the next section. Racemic [8]paracyclophane-10-methanol (±)-**19** was esterified with (−)-CSP acid **22**, yielding diastereomeric esters **20a** and **20b** (Figure 15a), which were not base-line separated in HPLC, as shown in Figure 15b [30]. However, by repeating HPLC, it was possible to separate them completely. Next, we tried the recrystallization of the obtained esters from various solvents, but in all cases both esters were obtained as fine needles, which were unsuitable for X-ray analysis. So, to determine the ACs of these compounds, the second-eluted ester **20b** was converted to camphorsultam amide, which was identical to amide (*S*)-**18B** (Figure 15c). The (*S*)-AC of **18B** was clearly determined by X-ray analysis as shown in Figure 14. So, the ACs of esters **20a** and **20b** were determined to be (*R*) and (*S*), respectively [30].

The first-eluted ester (*R*)-**20a** was converted to [8]paracyclophane-10-carboxylic acid (*R*)-(+)-**12** and then to the methyl ester (*R*)-(+)-[CD(+)247.7]-**21** (Figure 15d). The second-eluted ester (*S*)-**20b** was similarly converted to methyl ester (*S*)-[CD(−)247.2]-**21**. These results clearly indicated that the (*S*)-AC of [8]paracyclophane-10-carboxylic acid (+)-**12** previously assigned by chemical correlations requiring many steps was wrong and should be reversed (Figure 11) [30]. In addition, the AC of acid (−)-**15** was also reversed (Figure 11). Thus, it should be emphasized that to determine the ACs of chiral compounds, it is necessary to select more straightforward and reliable methods as exemplified here.

## 3. Use of Camphorsultam-Phthalic Acid (CSP Acid) for Alcohols—The CSP Acid Method

### Application to Alcohols with an Aromatic Group

(−)-Camphorsultam **6** was useful for the chiral resolution of racemic carboxylic acids and AC determination by X-ray crystallography, as explained in Section 2. The development of novel chiral molecular tools applicable to racemic alcohols was desired. For this purpose, we have developed novel chiral molecular tools, camphorsultam-phthalic acid (CSP acid) (−)-**22** and camphorsultam-dichlorophthalic acid (CSDP acid) (−)-**23** (Figure 16).

**Figure 17:**
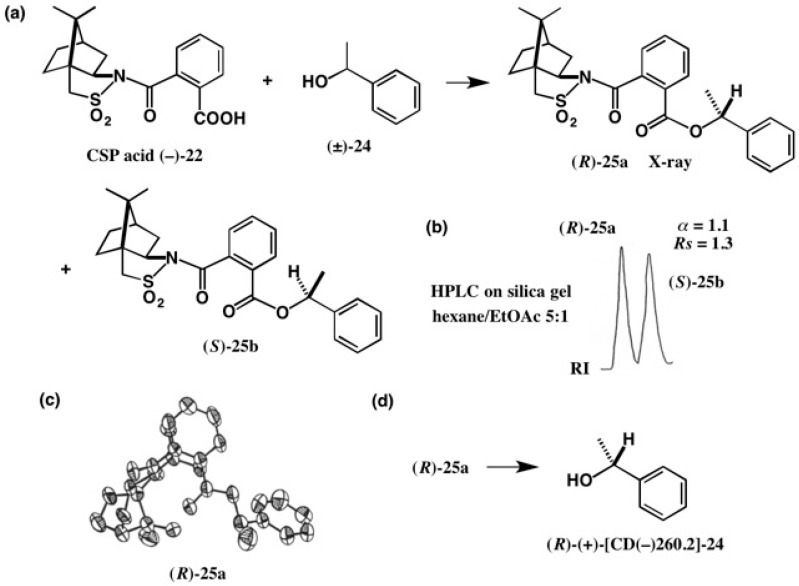
Preparation (**a**) and HPLC separation (**b**) of esters **25a** and **25b**, and the AC determination of ester (*R*)-**25a** by X-ray analysis (**c**). From ester (*R*)-**25a**, alcohol (*R*)-(+)-**24** was obtained (**d**). HPLC, redrawn from [32]. ORTEP drawing, reprinted from [31].

Camphorsultam-phthalic acid (−)-**22** was easily prepared by treating phthalic anhydride with the camphorsultam anion. The CSP acid method was first applied to a simple alcohol, as shown in Figure 17 [31]. Racemic 1-phenylethanol (±)-**24** was esterified with CSP acid (−)-**24** yielding diastereomeric esters **25a** and **25b**, which were separated well by HPLC on silica gel (α = 1.1, *Rs* = 1.3) (Figure 17b). The reason why we selected the phthalic acid as the connector between camphorsultam and acid moieties is that these two groups are close to each other, and hence the diastereomers are more different in stereochemistry and polarity, which would lead to larger separation in HPLC.

The first-eluted ester **25a** was recrystallized from MeOH giving prisms suitable for X-ray analysis. From the ORTEP drawing shown in Figure 17c, the AC of the 1-phenylethanol moiety was clearly determined as (*R*), because the AC of the CSP acid part was already known. The first-eluted ester (*R*)-**25a** was then converted to 1-phenylethanol (*R*)-(+)-**24**. The AC of the 1-phenylethanol previously assigned was thus corroborated by X-ray crystallography [31].

**Figure 18:**
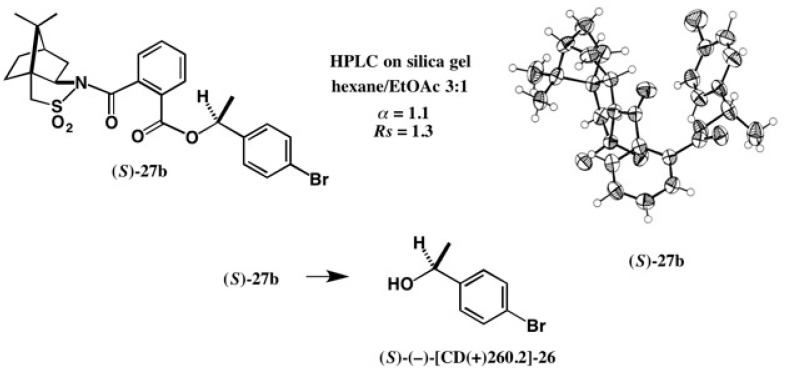
Preparation and HPLC separation of esters **27a** and **27b**, and the AC determination of ester (*S*)-**27b** by X-ray analysis. From ester (*S*)-**27b**, alcohol (*S*)-(−)-**26** was obtained. ORTEP drawing, reprinted from [31].

The CSP acid method was next applied to 1-(4-bromophenyl)ethanol **26** as shown in Figure 18 [31].

Racemic alcohol (±)-**26** was esterified with CSP acid (−)-**24** yielding diastereomeric esters **27a** and **27b**, which were similarly separated by HPLC on silica gel (α = 1.1, *Rs* = 1.3). Both esters **27a** and **27b** were recrystallized from MeOH giving prisms. A single crystal of **27b** was subjected to X-ray analysis giving the ORTEP drawing as shown in Figure 18, where the (*S*)-AC was unambiguously determined by the heavy atom effects of S and Br atoms (real image, *R* = 0.0352; mirror image, *R* = 0.0469). The (*S*)-AC was also confirmed by the internal reference method of AC using the camphorsultam unit. From ester **27b**, alcohol (*S*)-(−)-[CD(+)260.2]-**26** was recovered. The AC of alcohol **26** was thus established by X-ray crystallography [31].

**Figure 19:**
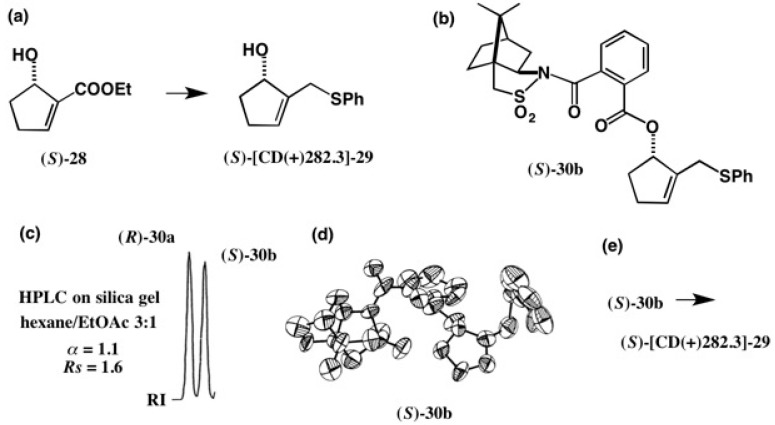
Preparation (**a**,**b**) and HPLC separation (**c**) of esters **30a** and **30b**, and the AC determination of ester (*S*)-**30b** by X-ray analysis (**d**). From ester (*S*)-**30b**, alcohol (*S*)-[CD(+)282.3]-**29** was obtained (**e**). HPLC, redrawn from [32]. ORTEP drawing, reprinted from [31].

The enzymatic method is also useful for the chiral resolution of racemates. For example, alcohol **28** was resolved by treating its racemic acetate with the lipase PS yielding chiral alcohol **28**, from which a chiral synthon **29** was prepared. However, the enantiomeric excess (ee) and AC of these compounds remained undetermined. So, the CSP acid method was applied to alcohol **29** as shown in Figure 19 [31].

The CSP esters **30a** and **30b** were almost baseline separated as shown in Figure 19c (α = 1.1, *Rs* = 1.6). The X-ray analysis of ester **30b** gave the ORTEP drawing (Figure 19d), where the final *R* value remained as high as *R* = 0.146 because of the poor crystallinity of ester **30b**. Therefore, its AC could not be determined by the heavy atom effect, but easily determined by the internal reference method of camphorsultam to be (*S*). Finally, ester (*S*)-**30b** was converted to alcohol (*S*)-[CD(+)282.3]-**29**, where [CD(+)282.3] indicates the enantiomer showing a positive CD Cotton effect at 282.3 nm [31]. To specify an enantiomer, CD data are useful because the CD measurements need a smaller amount of sample than that for the ${\left[\alpha \right]}_{D}$ measurement.

## 4. Camphorsultam-Dichlorophthalic Acid (CSDP Acid) for Alcohols—The CSDP Acid Method

As exemplified above, the CSP acid method is useful for the chiral resolution of racemic alcohols and the determination of ACs by X-ray crystallography. However, HPLC separation of diastereomeric CSP esters was not always effective, and in some cases, CSP esters crystallized as fine needles, which were not suitable for X-ray analysis. So, to improve the performance of the CSP acid method, we have explored the structure of chiral acids, and have found that the camphorsultam dichlorophthalic acid (CSDP acid) (−)-**23** (Figure 16) was much more useful than CSP acid (−)-**22**.

### 4.1. Synthesis of Chiral Molecular Motor

**Figure 20:**
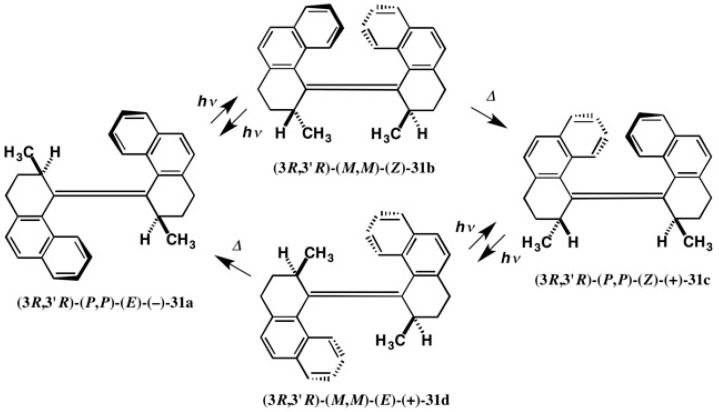
Light-powered molecular motor **31** and rotation mechanism, where **31a**–**31d** are rotation isomers. Redrawn with permission from [37].

Molecular machines are very interesting and attractive as future mechanical machines of molecular scale. We have developed the light–powered molecular motor **31a–31d** [33,34,35,36] as shown in Figure 20 [37], where *trans*-olefin **31a** isomerizes to *cis*-olefin **31b** under photo-irradiation. In this step, the left naphthalene moiety rotates counter-clockwise against the right naphthalene moiety. Furthermore, thermally unstable *cis*-olefin **31b** is converted to thermally stable *cis*-olefin **31c**, where the left naphthalene group again rotates counter-clockwise against the right naphthalene group. This thermal step is irreversible, although the photo-isomerization step is reversible, and hence in the steps, *trans*-olefin **31a** → *cis*-olefin **31b** → *cis*-olefin **31c**, the rotation occurs in a counter- clockwise manner [36,37].

Similarly, *cis*-olefin **31c** isomerizes to thermally unstable *trans*-olefin **31d** under photo-irradiation (Figure 20). In this step, the left naphthalene moiety again rotates counter-clockwise against the right naphthalene moiety. Furthermore, *trans*-olefin **31d** is converted to thermally more stable *trans*-olefin **31a**, where the left naphthalene group again rotates counter-clockwise against the right naphthalene group. This thermal step is again irreversible, although the photo-isomerization step is reversible, and hence the rotation occurs in a counter-clockwise manner in the steps, *cis*-olefin **31c** → *trans*-olefin **31d** → *trans*-olefin **31a**. The rotation occurs only in a counter-clockwise manner [36,37].

It should be emphasized that molecular motor **31** undergoes the photo and thermal reactions **31a** → **31b** → **31c** → **31d** → **31a**, where the left naphthalene group rotates counter-clockwise against the right naphthalene group. Therefore, by repeating these reactions, the molecular motor continuously rotates one-way [36,37]. It is obvious that the direction of motor rotation is governed by the molecular chirality, and hence it is important to synthesize the enantiopure molecular motor **31** and also to determine its absolute configuration in an unambiguous manner.

**Figure 21:**
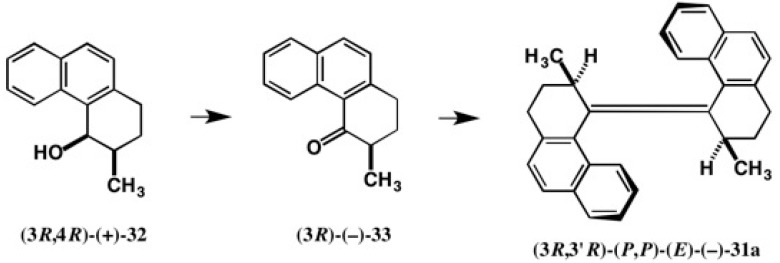
Synthesis of a chiral light-powered molecular motor (3*R*,3′*R*)-(*P*,*P*)-(*E*)-(−)-**31a** starting from alcohol (3*R*,4*R*)-(+)-**32**.

**Figure 22:**
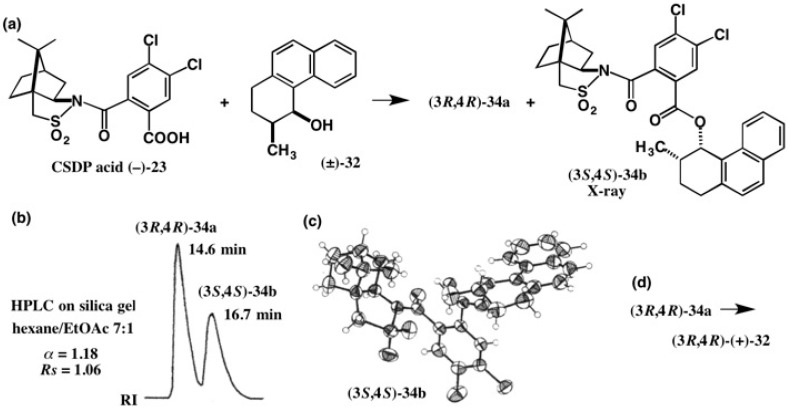
Preparation (**a**) and HPLC separation (**b**) of esters **34a** and **34b**, and the AC determination of ester **34b** by X-ray analysis (**c**). From ester (3*R*,4*R*)-**34a**, alcohol (3*R*,4*R)*-(+)-**32** was obtained (**d**). Redrawn from [38].

The molecular motor **31a** was synthesized in an enantiopure form, starting from alcohol (3*R*,4*R*)-(+)-**32** as shown in Figure 21 [33]. To obtain enantiopure alcohol **32** and to determine its AC, racemic *cis*-alcohol (±)-**32** was esterified with CSP acid (−)-**22**, and the obtained diastereomeric CSP esters were separated by HPLC on silica gel (α = 1.10). However, both CSP esters were obtained as fine needles or an amorphous solid. Hence their ACs could not be determined. So, we applied the new CSDP acid method, as shown in Figure 22 [33,38].

Racemic *cis*-alcoho l (±)-**32** was esterified with CSDP acid (−)-**23**, yielding diastereomeric CSDP esters, which were separated well by HPLC on silica gel (α = 1.18, *Rs* = 1.06) [38]. Thus, the HPLC separation of CSDP esters was better than that of CSP esters. Furthermore, the second-eluted CSDP ester **34b** was obtained as colorless prisms by recrystallizing from EtOAc, and hence the AC of CSDP ester **34b** could be determined to be (3*S*,4*S*) by X-ray crystallography using the heavy atom effects of S and the two Cl atoms (real image, *R* = 0.0287; mirror image, *R* = 0.0448). The (3*S*,4*S*) AC was also confirmed by the internal reference method of AC using the CSDP acid unit. Treatment of the first-eluted ester (3*R*,4*R*)-**34a** with LiAlH4 yielded the enantiopure alcohol (3*R*,4*R*)-(+)-**32**, from which molecular motor (−)-**31a** was synthesized as shown in Figure 21 and Figure 22 [33].

Motor compound (−)-**31a** was obtained as prisms by recrystallizing from MeOH, and a single crystal was subjected to X-ray crystallography. It was easy to determine the helicity of the molecular skeleton of (−)-**31a** to be (*P*,*P*) based on the AC of the methyl group position. Thus, the relative and absolute configurations of molecular motor (−)-**31a** were unambiguously determined to be (3*R*,3′*R*)-(*P*,*P*)-(*E*) [33].

### 4.2. Application to Diphenyl Methanols

The so-called asymmetric synthesis is very useful for the preparation of chiral compounds, as it is well known. However, there are some drawbacks in the asymmetric synthesis: (i) the products were not always enantiopure; (ii) the ACs were sometimes assigned in an empirical manner, e.g., comparison with the previous data of similar compounds, or assignment due to the asymmetric reaction mechanism. The next example emphasizes that the AC of products should be unambiguously determined by X-ray crystallography.

**Figure 23:**
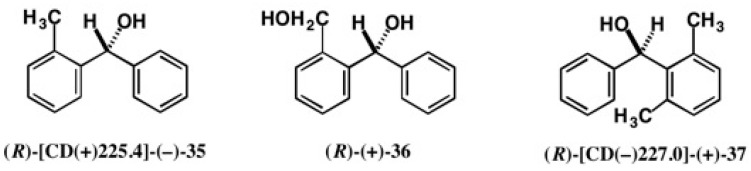
ACs of chiral *o*-substituted diphenylmethanols.

The AC of chiral alcohol **35**, (2-methylphenyl)phenylmethanol had been a source of much confusion (Figure 23). In 1967, Cervinka and coworkers reported it to be (*R*)-(−) based on the asymmetric reductions [39]. However, in 1985, Seebach and coworkers assigned it to be (*R*)-(+) by catalytic asymmetric reactions [40]. Which assignment is correct? To solve this problem, we have applied the CSDP acid method as follows.

**Figure 24:**
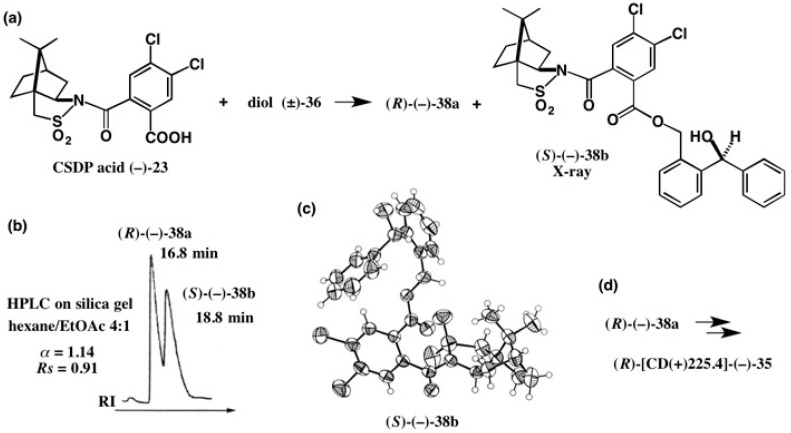
Preparation (**a**) and HPLC separation (**b**) of esters **38a** and **38b**, and the AC determination of ester (*S*)-(–)-**38b** by X-ray crystallography (**c**). Ester (*R*)-(–)-**38a** was converted to alcohol (*R*)-[CD(+)225.4]-(−)-**35** (**d**). HPLC, redrawn from [42]. ORTEP drawing, reprinted with permission from [41].

Racemic alcohol (±)-**35** was esterified with CSDP acid (−)-**23**, yielding diastereomeric CSDP esters. However, the diastereomeric esters appeared as a single peak in HPLC on silica gel; they could not be separated at all. So, we have tested some synthetic precursors of alcohol **35**, but all attempts were unsuccessful. Finally, we have found that diol **36** (Figure 23) could be separated as CSDP esters as shown in Figure 24 [41].

Diol (±)-**36** was esterified with CSDP acid (–)-**23**, yielding diastereomeric CSDP esters **38a** and **38b**, where the primary OH group was esterified. We had worried that the HPLC separation would become difficult, because in these esters the chiral groups in the CSDP acid moiety and alcohol part are remote from each other. However, the diastereomeric esters could be separated well by HPLC on silica gel, as shown in Figure 24b (α = 1.14, *Rs* = 0.91) [41].

The first-eluted ester, (−)-**38a**, was recrystallized from EtOH, giving prisms, one of which was subjected to X-ray analysis. However, it was found that the crystal was a twin, and hence it was unsuitable for X-ray crystallography. The second-eluted ester (−)-**38b** was similarly recrystallized from EtOH, giving single crystals, and the ORTEP drawing was obtained as shown in Figure 24c, where the (*S*)-AC was unambiguously determined by the Bijvoet method using one S and two Cl atoms (real image, *R* = 0.0324, *Rw* (weighted *R*-value) = 0.0415; mirror image, *R* = 0.0470, *Rw* = 0.0627). The (*S*)-AC was also corroborated by the internal reference method of AC using the CSDP acid unit. Starting from the first-eluted ester, (*R*)-(−)-**38a**, the desired alcohol (*R*)-[CD(+)225.4]-(−)-**35** was synthesized (Figure 24d). Thus, the (*R*)-AC of alcohol (−)-**35** has first been unambiguously determined by X-ray crystallography and chemical correlation [41]. It is again emphasized that the ACs of the asymmetric reaction products should be determined by an independent physical method such as X-ray crystallography.

**Figure 25:**
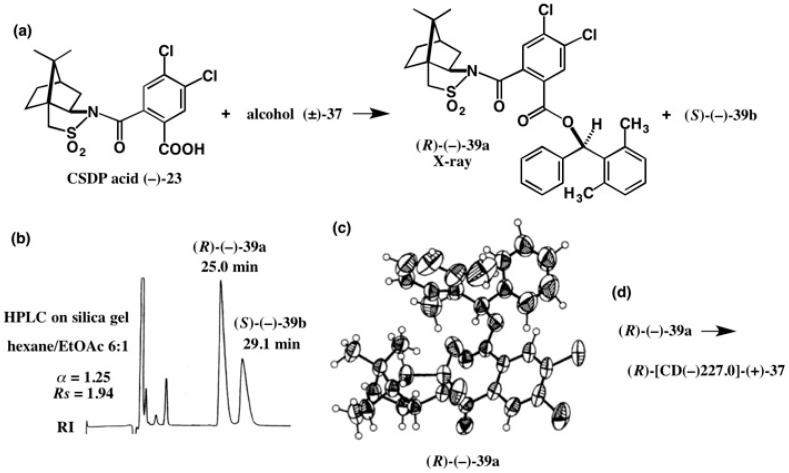
Preparation (**a**) and HPLC separation (**b**) of esters **39a** and **39b**, and the AC determination of ester (*R*)-(−)-**39a** by X-ray crystallography (**c**). Alcohol (*R*)-[CD(−)227.0]-(+)-**37** was recovered from ester (*R*)-(−)-**39a** (**d**). HPLC, redrawn from [44]. ORTEP drawing, reprinted from [43].

The next data provide the very interesting results and precautions for AC determination of similar and closely related compounds [43]. Chiral alcohol **37**, (2,6-dimethylphenyl)phenyl- methanol (Figure 23), is similar in structure to (2-methylphenyl)phenylmethanol **35**; the difference is one methyl or two methyl groups in the *o*-position. Therefore, we thought that the CSDP acid method would not be applicable to alcohol **37** directly, because CSDP esters of alcohol **35** could not be separated. However, it was surprising to find that CSDP esters **39a** and **39b** of alcohol **37** were base-line separated by HPLC on silica gel as shown in Figure 25b (α = 1.25, *Rs* = 1.94) [43].

**Figure 26:**
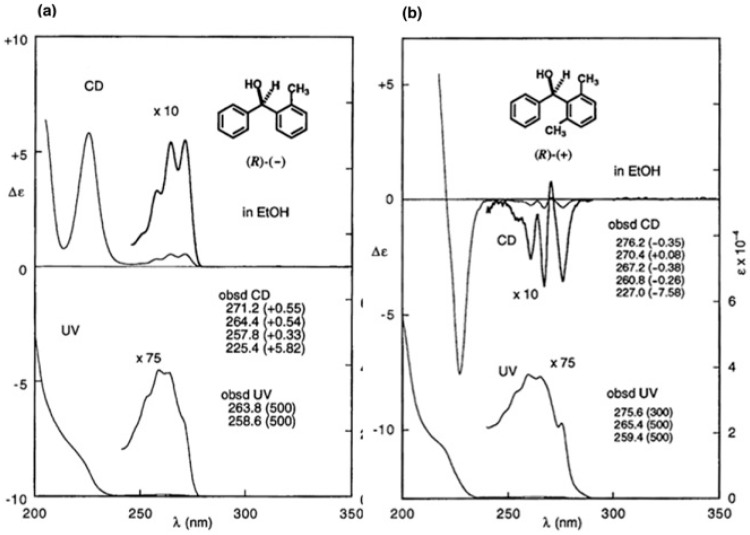
CD and UV spectra of *o*-substituted diphenylmethanols. (**a**) (2-methylphenyl)phenyl-methanol (*R*)-[CD(+)225.4]-(−)-**35**. Reprinted with permission from [41]; (**b**) (2,6-dimethylphenyl)-phenylmethanol (*R*)-[CD(−)227.0]-(+)-**37**. Reprinted from [43].

The first-eluted CSDP ester (−)-**39a** was converted to alcohol (+)-**37**, whose CD and UV spectra are shown in Figure 26b. It is interesting to see that the CD spectra of (2-methylphenyl)-phenylmethanol (*R*)-(−)-**35** and (2,6-dimethylphenyl)phenylmethanol (+)-**37** are similar in shape to each other, but opposite in sign (Figure 26). Therefore, we had once naturally thought that alcohol (+)-**37** would have the opposite AC, i.e., (*S*)-AC. However, it was surprising to find that the comparison of CD spectra leads to an erroneous AC assignment in this case [43], as will be explained below.

Later, we could obtain single crystals of the first-eluted ester (−)-**39a** by recrystallization from EtOH, and a crystal was subjected to X-ray crystallography. The ORTEP drawing is illustrated in Figure 25c, where (*R*)-AC was determined by the heavy atom effect (real image, *R* = 0.0358, *Rw* = 0.0488; mirror image, *R* = 0.0395, *Rw* = 0.0536) [43]. The (*R*)-AC was also confirmed by the internal reference method of AC using the CSDP acid unit. Since alcohol (+)-**37** was obtained from the first-eluted CSDP ester (*R*)-(−)-**39a**, (*R*)-AC was assigned to alcohol (+)-**37**. Thus, we could find that the AC assignment by comparison of CD spectra led to erroneous conclusions in this case [43]. X-ray crystallography using an internal reference of AC is thus very powerful for clear AC determination.

### 4.3. Synthesis of a Cryptochiral Hydrocarbon

**Figure 27:**
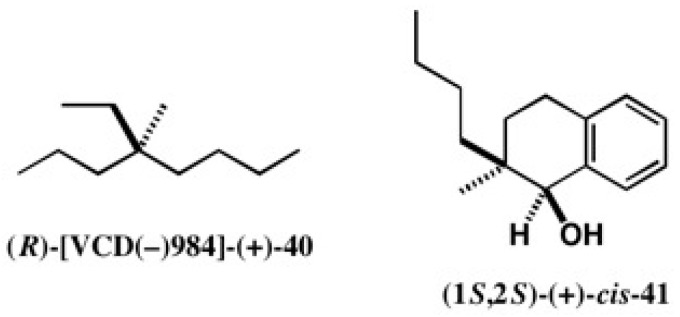
Cryptochiral hydrocarbon (*R*)-[VCD(−)984]-(+)-**40** and synthetic precursor (1*S*,2*S*)-(+)-*cis*-**41**.

4-Ethyl-4-methyloctane **40** can exist as a basic and simple chiral hydrocarbon, where methyl, ethyl, propyl, and butyl groups are connected to a quaternary chiral center (Figure 27). Since the optical rotation value of **40** is very small as ${\left[\alpha \right]}_{D}$ = +0.19 (neat), it may be called a cryptochiral hydrocarbon [45].

In 1980, H. Wynberg and a coworker first synthesized chiral hydrocarbon (−)-**40** (95% ee), but its AC could not be determined [46]. In 1988, L. Lardicci and coworkers reported the synthesis and determination of the AC of (+)-**40**, where they applied the CD exciton chirality method to acetylene tertiary alcohol benzoate for determining AC, but its observed CD was very weak (λ_ext_ = 239 nm, ∆ε = +0.8) [47]. Therefore, it is difficult to say that the AC was unambiguously determined.

**Figure 28:**
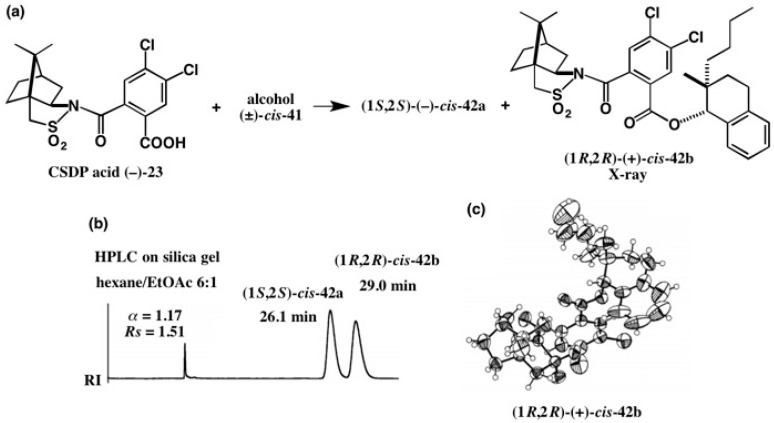
Preparation (**a**) and HPLC separation (**b**) of CSDP esters **42a** and **42b**, and the AC determination of ester (1*R*,2*R*)-(+)-*cis*-**42b** by X-ray crystallography (**c**). HPLC and ORTEP, reprinted with permission from [45].

We selected the alcohol *cis*-**41** as a synthetic precursor of hydrocarbon **40** (Figure 27). To obtain enantiopure alcohol *cis*-**41** and to determine its AC, the CSDP acid method was applied, as shown in Figure 28 [45]. Racemic alcohol (±)-*cis*-**41** was esterified with CSDP acid (−)-**23**, yielding diastereomeric esters **42a** and **42b**, which were baseline separated by HPLC on silica gel as shown in Figure 28b [45]. The second-eluted ester **42b** was recrystallized from EtOH/CH_2_Cl_2_ giving single crystals, one of which was subjected to X-ray analysis. Figure 28c shows the ORTEP drawing of **42b**, where AC was determined by the heavy atom effect (real image, *R* = 0.0598, *Rw* = 0.0740; mirror image, *R* = 0.0719, *Rw* = 0.0899). The (1*R*,2*R*)-AC of ester **42b** was also established by the internal reference method of AC using the CSDP acid unit [45].

**Figure 29:**
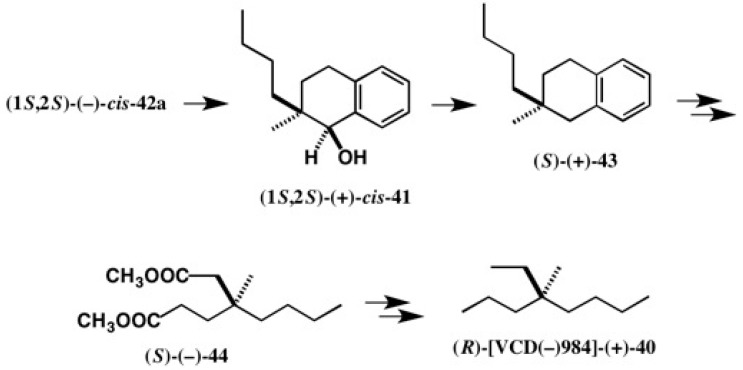
Synthesis of cryptochiral hydrocarbon (*R*)-[VCD(−)984]-(+)-**40** starting from CSDP ester (1*S*,2*S*)-(−)-*cis*-**42a**.

Starting from the first-eluted ester (1*S*,2*S*)-(−)-*cis*-**42a**, enantiopure cryptochiral hydrocarbon (*R*)-[VCD(−)984]-(+)-**40** was synthesized as shown in Figure 29, where [VCD(−)984] indicates an enantiomer showing a negative vibrational circular dichroism (VCD) band at 984 cm^−1^ [45]. It should be noted that the (*R*)-AC of [VCD(−)984]-**40** was also determined by the quantum mechanical calculation of VCD and IR, where the theoretically calculated VCD and IR spectra were compared with the observed spectra [48]. The theoretical AC determination was thus established in an experimental manner, as explained here.

### 4.4. Application to 2-(1-Naphthyl)Propane-1,2-Diol

**Figure 30:**
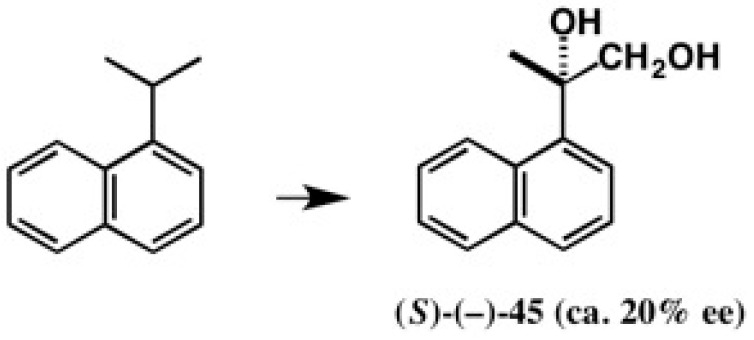
Biotransformation of 1-*iso*-propylnaphthalene to 2-(1-naphthyl)propane-1,2-diol **45**.

It was previously reported that 1-*iso*-propylnaphthalene was biotransformed in rabbits to 2-(1-naphthyl)propane-1,2-diol (*S*)-(−)-**45** (Figure 30) [49]. However, its enantiomeric excess was very low (ca. 20% ee) and its AC was determined by chemical correlation to a compound, the AC of which had been determined by an empirical rule. Therefore, it was desired to obtain enantiopure diol **45** and to determine its AC in an unambiguous manner.

**Figure 31:**
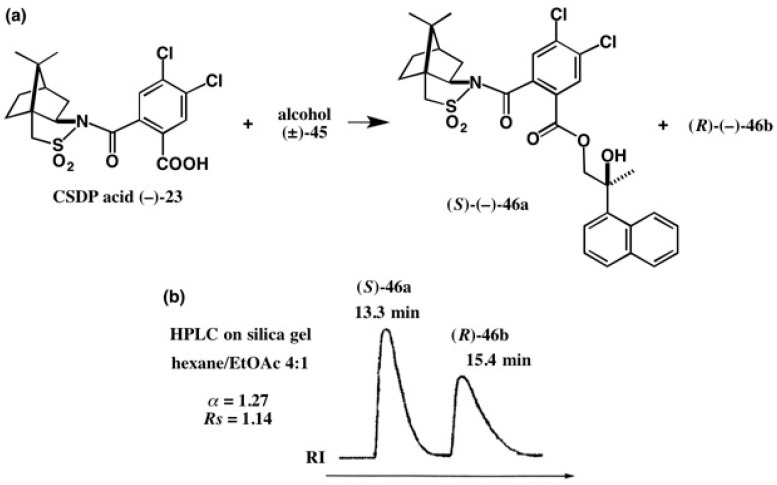
Preparation (**a**) and HPLC separation (**b**) of CSDP esters **46a** and **46b**. HPLC, reprinted from [42].

We have applied the CSDP acid method to racemic diol **45**, as illustrated in Figure 31 [50]. Although these are CSDP esters of primary alcohols, where the chiral moiety of the alcohol part is far from that of the CSDP acid part, these diastereomers were largely separated by HPLC on silica gel (separation factor α = 1.27) [50]. This large α value indicates that esters **46a** and **46b** were more efficiently separated than the other CSDP esters discussed above. It may be due to the free OH group.

**Figure 32:**
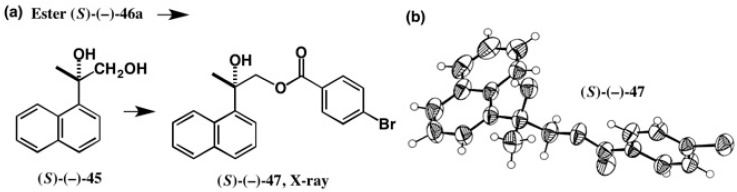
Preparation (**a**) of *p*-bromobenzoate (*S*)-(−)-**47** and its X-ray analysis (**b**). ORTEP drawing, reprinted from [50].

To obtain single crystals suitable for X-ray analysis, we tried to recrystallize these esters from various solvents, but all attempts were unsuccessful. So, we have adopted the following strategy, as shown in Figure 32 [50].

The reduction of the first-eluted ester **46a** with LiAlH4 and successive benzoylation with *p*-Br-BzCl furnished *p*-bromobenzoate (−)-**47**, which was recrystallized from EtOH, giving good single crystals suitable for X-ray crystallography. The ORTEP drawing of (−)-**47** is shown in Figure 32b, where the (*S*)-AC was unambiguously determined by the original Bijvoet method. Namely, the 18 Bijvoet pairs were observed, and their observed intensity ratios agreed well with the calculated ratios [50]. In addition, the final *R*-value also indicated the (*S*)-AC (real image, *R* = 0.0249, *Rw* = 0.0342; mirror image, *R* = 0.0358, *Rw* = 0.0520). From these results, the (*S*)-AC of 2-(1-naphthyl)propane-1,2-diol (−)-**45** was established.

## 5. Use of 2-Methoxy-2-(1-Naphthyl)Propionic Acid (MαNP Acid) for Alcohols—The MαNP Acid Method

### 5.1. Synthesis of Enantiopure MαNP Acid, HPLC Separation, and AC Determination by ^1^H-NMR Diamagnetic Anisotropy

**Figure 33:**
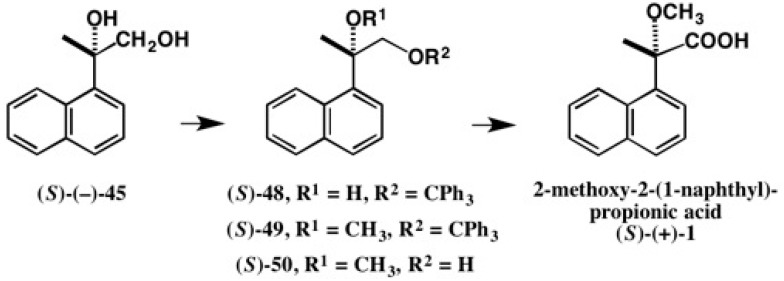
Conversion of diol (*S*)-(−)-**45** to 2-methoxy-2-(1-naphthyl)propionic acid (*S*)-(+)-**1**.

During the AC determination of diol (−)-**45**, we realized that it was possible to synthesize enantiopure 2-methoxy-2-(1-naphthyl)propionic acid **1**, and the conversion was actually carried out as shown in Figure 33 [51]. Starting from enantiopure diol (*S*)-(−)-**45**, enantiopure 2-methoxy- 2-(1-naphthyl)propionic acid (*S*)-(+)-**1** was obtained, indicating that the (*S*)-AC of acid (+)-**1** was established.

**Figure 34:**
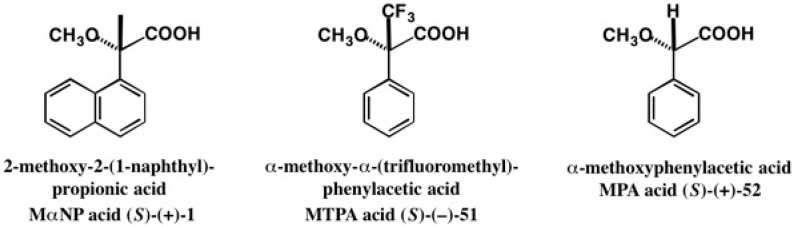
Chiral acids useful for the ^1^H-NMR diamagnetic anisotropy method.

Chiral acid **1** is similar in structure to the Mosher’s α-methoxy-α-(trifluoromethyl)phenylacetic acid (MTPA acid) **51** [52,53] and also to α-methoxyphenylacetic acid (MPA acid) **52** [54] (Figure 34). The NMR methods using these chiral acids have been widely employed for determining the ACs of various chiral secondary alcohols including natural products by using ^1^H-NMR diamagnetic anisotropy shift data, although these methods are empirical rules. So, we had expected that acid **1** was also useful for determining the ACs of chiral secondary alcohols by the ^1^H-NMR diamagnetic anisotropy method.

**Figure 35:**
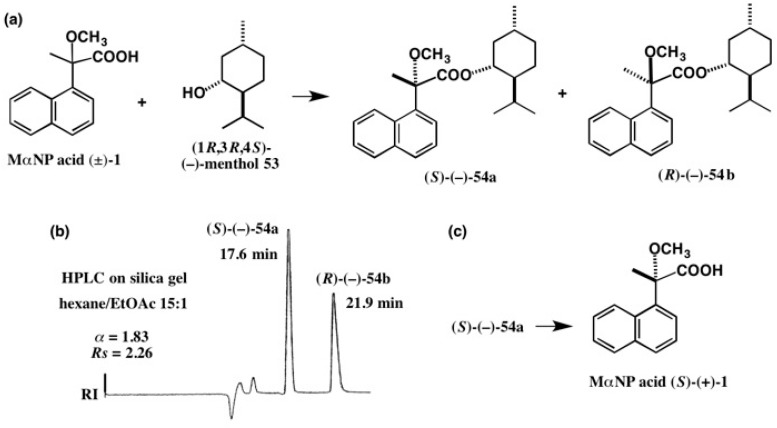
Preparation (**a**) and HPLC separation (**b**) of menthol MαNP esters **54a** and **54b**. (**c**) Recovery of MαNP acid (*S*)-(+)-**1** from the first eluted ester (−)-**54a**. HPLC, reprinted with permission from [51].

To employ as a chiral shift reagent, it was necessary to prepare enantiopure acid **1** on a large scale. So, we had looked for a simpler method to enantioresolve racemic acid (±)-**1**. It was surprising to find that natural menthol (−)-**53** was very useful for the chiral resolution of racemic acid **1**. Namely, racemic MαNP acid (±)-**1** was reacted with menthol (−)-**53** yielding diastereomeric esters **54a** and **54b**, which were largely separated by HPLC on silica gel (α = 1.83, *Rs* = 2.26) (Figure 35) [51]. Such a large separation factor α has never been observed in the previous CSP and CSDP esters. The first-eluted ester (−)-**54a** was treated with NaOCH_3_ in MeOH and then with water yielding MαNP acid (+)-**1**, which was identical to acid (*S*)-(+)-**1** shown in Figure 33. Therefore, the AC of the first-eluted ester (−)-**54a** was determined to be (*S*) [51].

**Figure 36:**
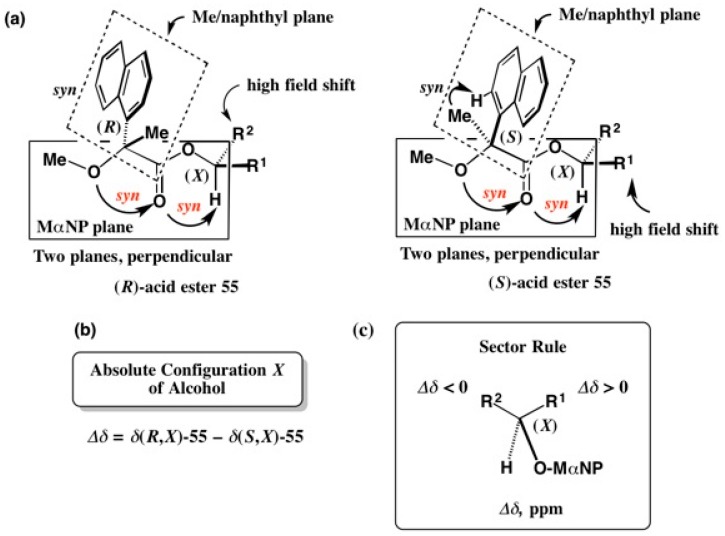
AC determination and mechanism of the ^1^H-NMR diamagnetic anisotropy method using (*R*)- and (*S*)-MαNP acids. (**a**) Preferred conformation of MαNP esters; (**b**) Definition of *Δδ*; (**c**) Sector rule for determining the absolute configuration. Redrawn with permission from [55].

Figure 36 shows the AC determination of a secondary alcohol by the ^1^H-NMR diamagnetic anisotropy method using (*R*)- and (*S*)-MαNP acids [55]. The (*R*)-acid ester **55** takes a preferred conformation as shown in Figure 36a, where the substituent R^2^ is located above the naphthalene ring, and hence it shows a high field shift.

On the other hand, the (*S*)-acid ester **55** takes a similar preferred conformation, as shown in Figure 36a. However, the substituent R^1^ shows a high field shift because it is located above the naphthalene ring. The chemical shift difference ∆δ is defined as ∆δ = δ(*R*,*X*)-**55** − δ(*S*,*X*)-**55**, where *X* denotes the AC of the alcohol part to be determined. From the observed ^1^H-NMR spectra, ∆δ values are calculated for each proton, and are plotted in the sector rule shown in Figure 36c, where the substituent R^1^ showing positive ∆δ values is placed on the right side. On the other hand, the substituent R^2^ showing negative ∆δ values is placed on the left side. Based on these data, the AC (*X*) of the alcohol part can be determined [55].

**Figure 37:**
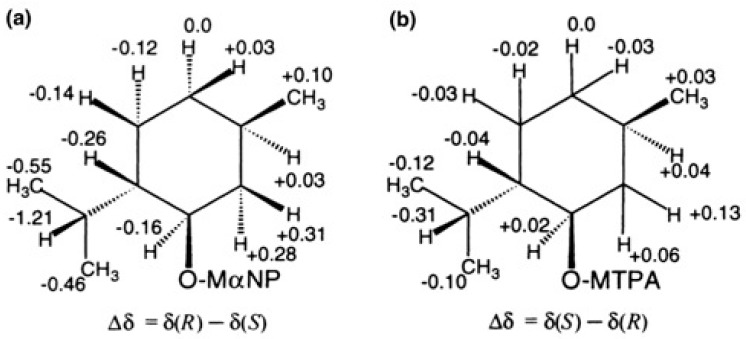
Distribution of ∆δ values in ppm and AC determination. (**a**) Menthol MαNP esters and (**b**) menthol MTPA esters. Reprinted with permission from [51].

Figure 37a shows the distribution of ∆δ values of menthol MαNP esters (*R*)-(−)-**54b** and (*S*)-(−)-**54a**, where the isopropyl group showing negative ∆δ values is placed on the left side, while the methyl group showing positive ∆δ value is placed on the right side. Based on these data, the AC of (−)-menthol was determined as shown in Figure 35 [51]. Of course, this AC agreed with the previously established AC of (−)-menthol.

Figure 37b shows the distribution of ∆δ values of menthol MTPA esters. It should be noted that ∆δ is defined as ∆δ = δ(*S*,*X*) − δ(*R*,*X*). From the distribution of ∆δ values, the AC of (−)-menthol was assigned as shown. However, compared with the data of Figure 37a,b, the ∆δ values of MαNP esters are much larger than those of MTPA esters; ca. four times larger [51]. This is a great advantage of the MαNP ester method.

**Figure 38:**
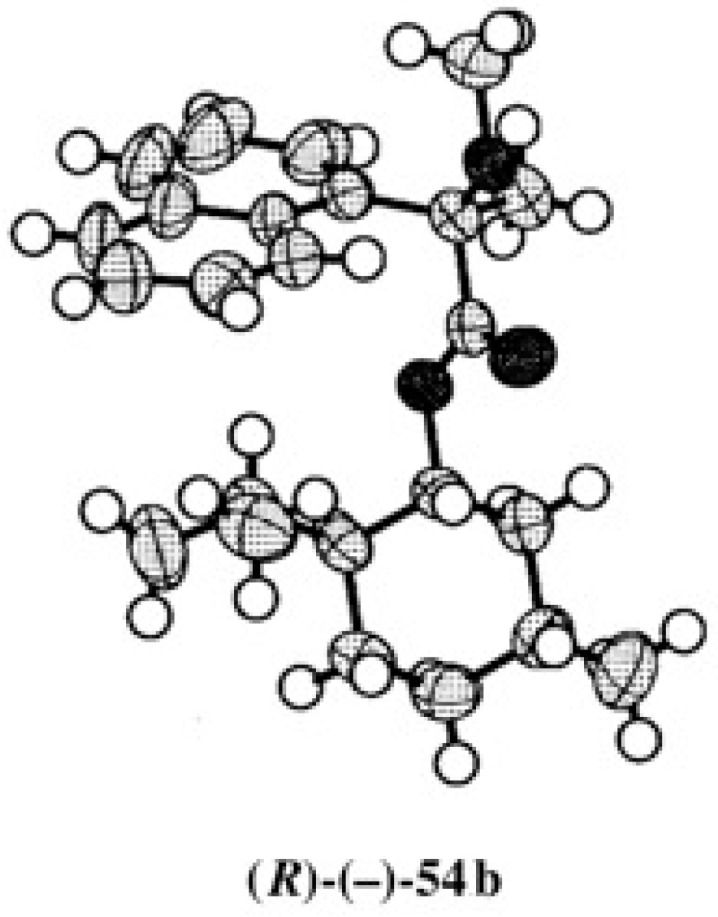
X-ray ORTEP drawing of menthol MαNP ester (*R*)-(−)-**54b**. Reprinted with permission from [56].

It was difficult to obtain single crystals of menthol MαNP esters **54a** and **54b**, although we had tried many recrystallizations from various solvents. Finally, we could obtain single crystals of ester (−)-**54b** suitable for X-ray analysis by recrystallizing from a mixed solvent (Et_2_O/MeOH). From the ORTEP drawing, the (*R*)-AC of the MαNP acid moiety was established (Figure 38) [56]. The chemical correlation, ^1^H-NMR spectra, and X-ray analysis are thus consistent with each other.

### 5.2. Application of the MαNP Acid Method to Aromatic Alcohol

**Figure 39:**
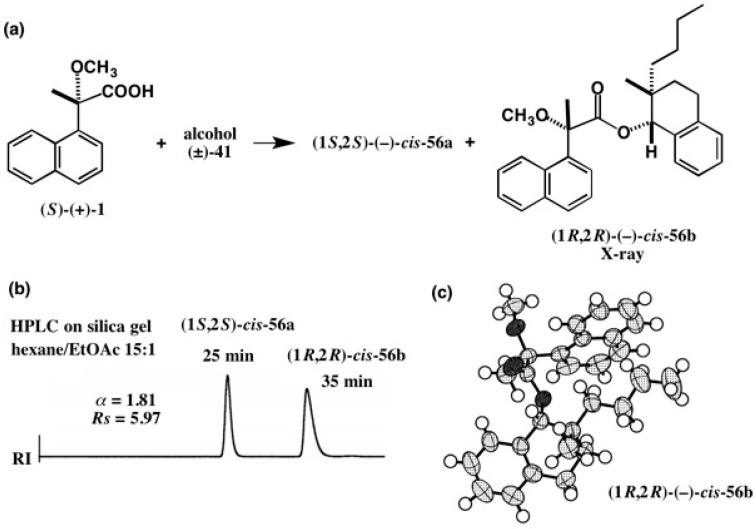
Preparation (**a**) and HPLC separation (**b**) of MαNP esters **56a** and **56b**. HPLC, reprinted with permission from [45]. (**c**) X-ray ORTEP drawing of MαNP ester (1*R*,2*R*)-(−)-*cis*-**56b**, reprinted with permission from [57].

As explained in Figure 35, one of the advantages of the MαNP acid method is that diastereomeric esters are largely separated by HPLC on silica gel. This was also proved by the example shown in Figure 39. Alcohol (±)-**41**, a synthetic precursor of a cryptochiral hydrocarbon **40** (Figure 27), was esterified with MαNP acid (*S*)-(+)-**1**, yielding diastereomeric esters **56a** and **56b**, which were largely separated by HPLC on silica gel (α = 1.81, *Rs* = 5.97) [45]. This is very remarkable, when compared with the case of CSDP acid esters shown in Figure 28, where α = 1.17, and *Rs* = 1.51. The (1*R*,2*R*)-AC of (−)-*cis*-**56b** was also confirmed by X-ray crystallography as shown in Figure 39c [57].

### 5.3. Application of Aliphatic Chain Alcohols

**Figure 40:**
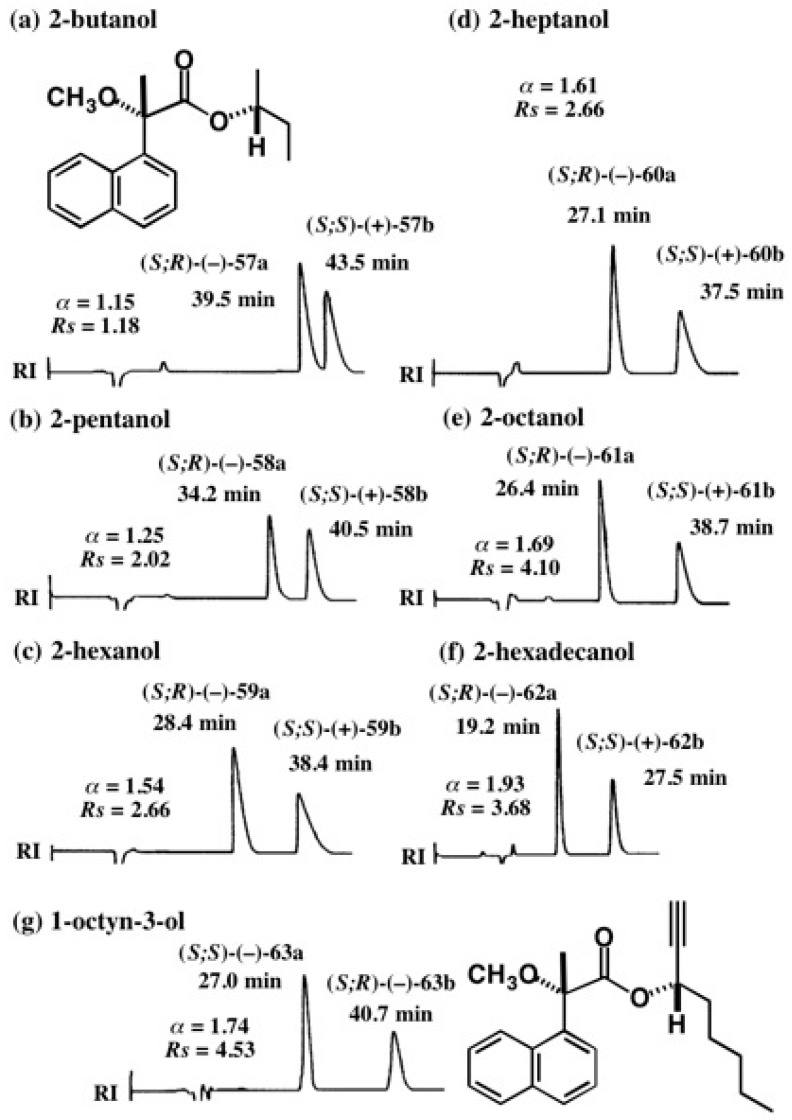
HPLC separation of diastereomeric MαNP esters of aliphatic linear alcohols and acetylene alcohol (**a**–**g**). Reprinted with permission from [58].

Another advantage of the MαNP acid method is that aliphatic chain alcohols can be resolved as MαNP esters, as exemplified in Figure 40 [58]. For example, racemic 2-butanol was esterified with (*S*)-(+)-MαNP acid yielding diastereomeric esters, which were almost baseline separated as shown in Figure 40a (α = 1.15, *Rs* = 1.18). In this case, the chirality of 2-butanol, i.e., the difference between methyl and ethyl groups, was recognized well by the MαNP acid/HPLC.

In the case of 2-pentanol, the chirality is made by the difference between methyl and propyl groups. The difference is larger than the difference between methyl and ethyl groups. Therefore, 2-pentanol MαNP esters are more largely separated by HPLC on silica gel as shown in Figure 40b (α = 1.25, *Rs* = 2.02) [58]. Thus, the HPLC separation reflects the difference of chain length.

This tendency becomes remarkable, when extending to longer chain alcohols [58], as seen in Figure 40; (c) 2-hexanol, difference between methyl and butyl groups: α = 1.54, *Rs* = 2.66; (d) 2-heptanol, difference between methyl and pentyl groups: α = 1.61, *Rs* = 2.66; (e) 2-octanol, difference between methyl and hexyl groups: α = 1.69, *Rs* = 4.10; (f) 2-hexadecanol, difference between CH_3_ and CH_3_(CH_2_)_13_ groups: α = 1.93, *Rs* = 3.68. Thus, HPLC separation is very sensitive to the difference of chain length.

**Figure 41:**
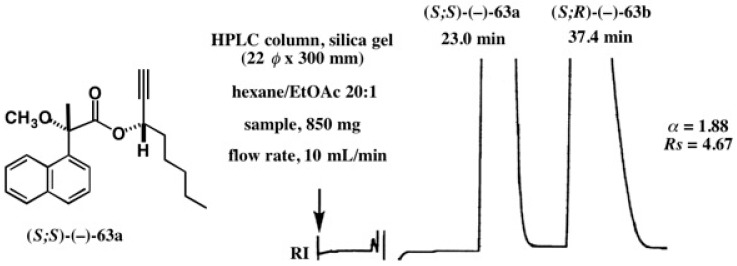
Preparative HPLC separation of 1-octyn-3-ol MαNP esters **63a**/**63b**: silica gel column, theoretical plate number, *n* = 9500–11,600; sample (850 mg) was injected. Reprinted with permission from [59].

The case of 1-octyn-3-ol is also unique, and diastereomers **63a**/**63b** were largely separated (α = 1.74, *Rs* = 4.53) (Figure 40g) [58]. Thus, the separation factor α of esters **63a**/**63b** is larger than that of 2-heptanol esters **60a**/**60b**, implying that the acetylene group is effective for HPLC separation. Such a large α value enabled HPLC separation on a large scale as shown in Figure 41, where an 850 mg sample was injected [59]. There is still enough space between the two bands, and hence it would be possible to load a larger amount of the sample. Thus, the MαNP acid method is very useful for the synthesis of enantiopure 1-octyn-3-ol on a large scale, which would be useful as a chiral synthon.

**Figure 42:**
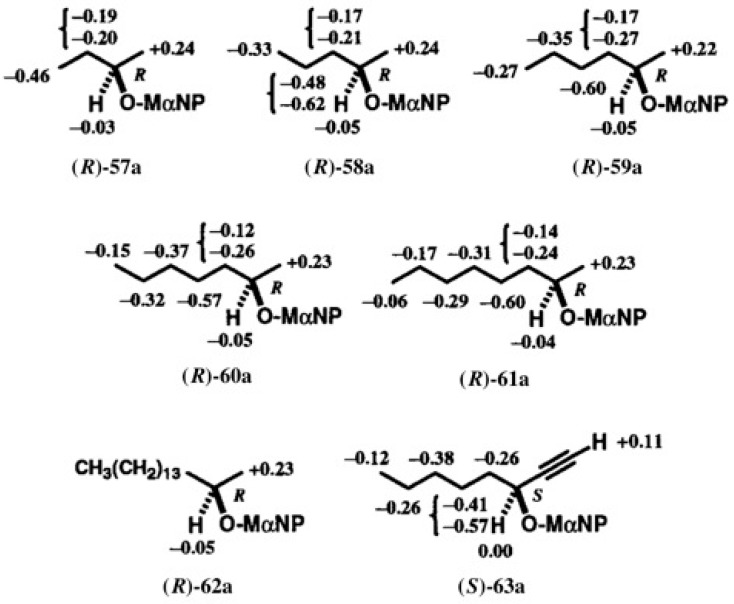
^1^H-NMR ∆δ values in ppm and ACs of the first-eluted MαNP esters. Reprinted with permission from [58].

To determine the ACs of these chain alcohol MαNP esters, ^1^H-NMR spectra were measured, and their ∆δ values were calculated as shown in Figure 42 [58]. In the case of MαNP esters, the chemical shift difference ∆δ is originally defined as ∆δ = δ(*R*;*X*) − δ(*S*;*X*), where *R* and *S* indicate the ACs of the MαNP acid part, while *X* indicates the AC of the alcohol moiety to be determined.

In Figure 1, Figure 39, and Figure 40, racemic alcohols were esterified with (*S*)-(+)-MαNP acid **1**. In such a case, the formula of ∆δ is transformed as follows. For example, in the case of 2-butanol, the first-eluted ester is defined as (*S*;*X*)-**57a**, where *X* denotes the AC of the alcohol part in the first-eluted MαNP ester. In addition, the second-eluted ester can be defined as (*S*;−*X*)-**57b**, where −*X* denotes the opposite AC of *X*. It should be noted that esters (*S*;−*X*) and (*R*;*X*) are enantiomers, and therefore, their chemical shift data are equal to each other. So, ∆δ = δ(*R*;*X*) − δ(*S*;*X*) = δ(*S*;−*X*) − δ(*S*;*X*) = δ(second-eluted ester) − δ(first-eluted ester) (see Figure 3). Based on this definition, ∆δ values were calculated [58].

In the case of 2-butanol esters **57a**/**57b**, the methyl group showed a positive ∆δ value, and hence was placed on the right side, while the ethyl group showed negative ∆δ values, and hence was placed on the left side. Therefore, the AC of the first-eluted ester was determined to be (*R*) (Figure 42). In other esters **58**–**62**, ∆δ values are reasonably distributed indicating (*R*)-ACs (Figure 42). Thus in the case of all MαNP esters **57**–**62**, the (*S*;*R*)-diastereomers were eluted first [58].

In the case of 1-octyn-3-ol MαNP esters **63a**/**63b**, the acetylene proton showed a positive ∆δ value, while the pentyl group showed negative ∆δ values, leading to the (*S*)-AC (Figure 42) [58].

### 5.4. Application of Acetylene Chain Alcohols

**Figure 43:**
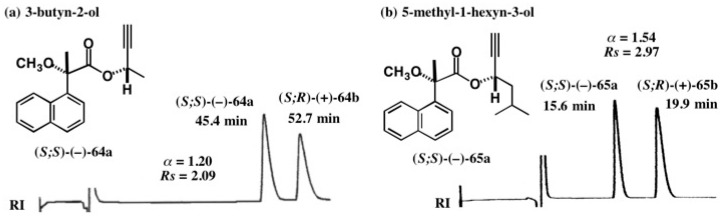
HPLC separation of diastereomeric MαNP esters of acetylene alcohols: (**a**) 3-butyn-2-ol, hexane/EtOAc 20:1, reprinted with permission from [60]; (**b**) 5-methyl-1-hexyn-3-ol, hexane/EtOAc 10:1, reprinted from [61].

The MαNP acid method has been applied to other acetylene alcohols as shown in Figure 43. In the case of 3-butyn-2-ol, the diastereomers **64a**/**64b** were separated well as shown in figure 42a (α = 1.20, *Rs* = 2.09) [60]. The result indicated that acetylene and methyl groups are recognized well by HPLC on silica gel. In the case of 5-methyl-1-hexyn-3-ol, the *iso*-butyl group is longer than the methyl group, and hence esters **65a**/**65b** were much more largely separated (α = 1.54, *Rs* = 2.97) [60].

**Figure 44:**
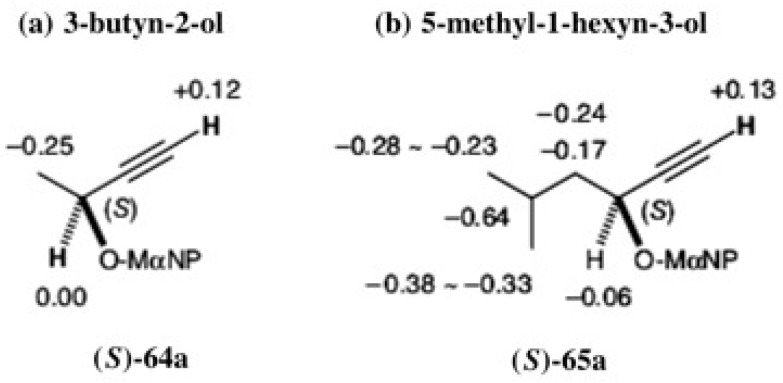
^1^H-NMR ∆δ values in ppm and ACs of the first-eluted MαNP esters **64a** (**a**) and **65a** (**b**). Reprinted with permission from [60].

It was easy to determine the ACs of these esters by applying the MαNP ester diamagnetic anisotropy method as shown Figure 44 [60]. In both cases, acetylene protons showed positive ∆δ values, while methyl and *iso*-butyl groups showed negative ∆δ values. Therefore, the ACs of the first-eluted esters were unambiguously determined to be (*S*).

**Figure 45:**
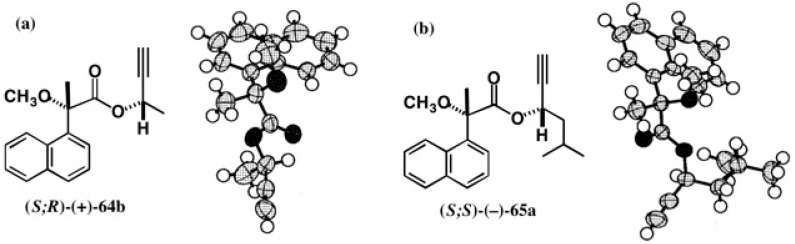
X-ray ORTEP drawings of MαNP esters (*S*;*R*)-(+)-**64b** (**a**) and (*S*;*S*)-(−)-**65a** (**b**). Reprinted with permission from [56].

It should be noted that the ACs of these MαNP esters could be confirmed by X-ray crystallography. We were lucky to obtain single crystals of MαNP esters (*S*;*R*)-(+)-**64b** and (*S*;*S*)-(−)-**65a**, when recrystallizing from MeOH. The ORTEP drawings are illustrated in Figure 45, where the ACs of the alcohol parts were clearly determined by using the MαNP acid part as an internal reference of AC [56]. The ACs determined by the ^1^H-NMR diamagnetic anisotropy method were thus corroborated by X-ray crystallography.

The ^1^H-NMR diamagnetic anisotropy method using MαNP acid has been originally developed as an empirical rule. However, we have never encountered any exception, where the AC determined by the ^1^H-NMR MαNP ester method disagreed with that by X-ray crystallography. This fact is very important for making the ^1^H-NMR MαNP ester method more reliable.

**Figure 46:**
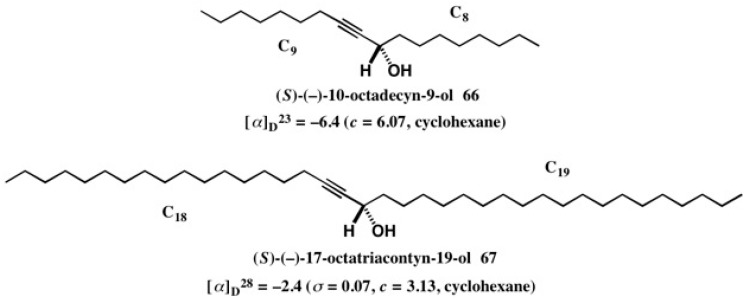
Enantiopure acetylene alcohols with established ACs, which were prepared by the MαNP acid method.

**Figure 47:**
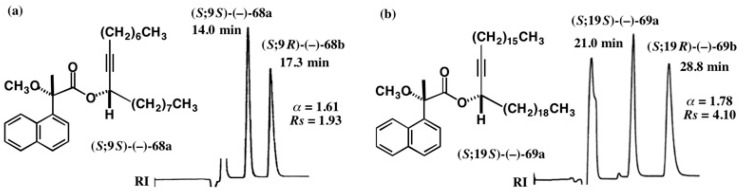
HPLC separation of diastereomeric MαNP esters of long chain internal acetylene alcohols: (**a**) **68a**/**68b**, hexane/EtOAc 20:1, reprinted from [61]; (**b**) **69a**/**69b**, hexane/EtOAc 50:1, reprinted with permission from [62].

The MαNP acid method is also applicable to internal acetylene alcohols with long chains, and some enantiopure alcohols with established ACs were synthesized as exemplified in Figure 46 [62]. Racemic acetylene alcohol (±)-**66** was esterified with (*S*)-(+)-MαNP acid yielding diastereomeric esters **68a**/**68b**, which were largely separated by HPLC on silica gel (α = 1.61, *Rs* = 1.93) (Figure 47a) [62]. It was surprising to find that although the two side chains are similar in length, i.e., C_8_ and C_9_, the diastereomers were well separated. This fact implies that the acetylene group makes a dominant contribution for HPLC separation.

The HPLC data of esters **69a**/**69b** are also interesting, because the separation factor α became larger (α = 1.78, *Rs* = 4.10) (Figure 47b) [62]. The two side chains consist of C_18_ and C_19_ and hence they are more similar to each other than in the case of **68a**/**68b**. However, the separation factor α is larger than that of **68a**/**68b**. This fact again indicates that the acetylene group is a key factor to control the HPLC separation on silica gel.

**Figure 48:**
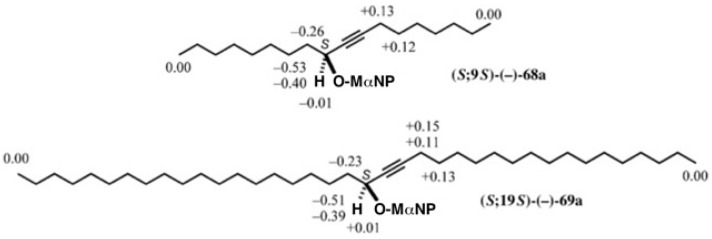
^1^H-NMR ∆δ values in ppm and ACs of the first-eluted MαNP esters **68a** and **69a**. Reprinted with permission from [62].

To determine the ACs of these compounds, ^1^H-NMR ∆δ values were calculated as shown in Figure 48 [62].

In MαNP esters **68a**/**68b**, the side chain containing the acetylene group showed positive ∆δ values, while the saturated side chain showed negative ∆δ values. Therefore, the AC of the first-eluted MαNP ester (−)-**68a** was determined to be (*S*). The same was found in MαNP esters **69a**/**69b**, which led to the (*S*)-AC of the first eluted ester (−)-**69a**.

**Figure 49:**
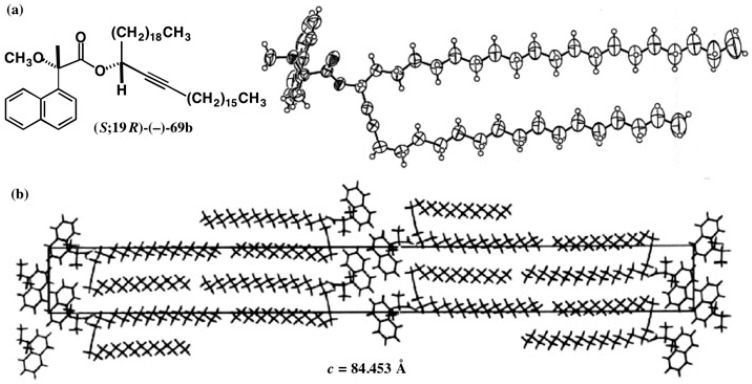
X-ray crystal structure of MαNP ester (*S*;19*R*)-(−)-**69b**. (**a**) ORTEP drawing; (**b**) Crystal packing of (*S*;19*R*)-(−)-**69b**: view along the *a* axis; the rectangle indicates a unit lattice. Reprinted with permission from [62].

MαNP esters **68a**, **68b**, and **69a** were obtained as a syrup or amorphous solid except ester **69b**, which was recrystallized from *iso*-PrOH, giving thin plate crystals. However, because their thickness was ca. 5 μm, a conventional X-ray machine could not be used. Instead, the strong X-ray of synchrotron radiation in the SPring-8 in Hyogo, Japan was used for X-ray crystallography (final *R* = 0.0814). The ORTEP drawing was obtained as shown in Figure 49a, where (19*R*)-AC was unambiguously determined by using the MαNP acid part as an internal reference [62]. Thus, the AC assigned by the ^1^H-NMR diamagnetic anisotropy was confirmed by X-ray crystallography.

Figure 49b shows the crystal packing of MαNP ester molecules **69b**, where the rectangle shows a unit lattice containing four molecules [62]. It is interesting that two side chains of a molecule are placed in parallel, and that these two alkyl chains form a pair with those of the second molecule in the unit lattice. These pairs are arranged so as to form an aliphatic bilayer in the crystal. The third and fourth molecules similarly form an aliphatic bilayer. It is very interesting that these aliphatic bilayer structures are similar to those of cell membranes.

**Figure 50:**
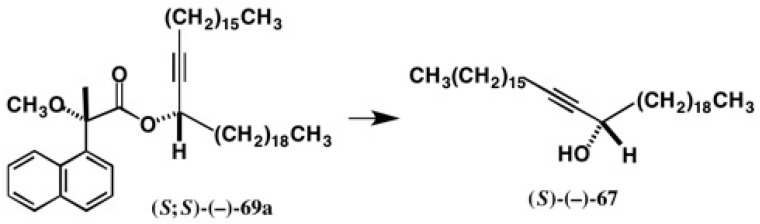
Preparation of enantiopure acetylene alcohol (*S*)-(−)-**67**.

It was easy to recover enantiopure long chain acetylene alcohol (*S*)-(−)-**67**, as shown in Figure 50. The specific rotation value of alcohol (*S*)-(−)-**67** was small, but it was still measurable as listed in Figure 46, where the observed ${\left[\alpha \right]}_{D}$ was larger than the standard deviation σ [62].

### 5.5. Synthesis of Enantiopure Saturated Long Chain Alcohol with Established AC

Next we tried to synthesize enantiopure saturated long chain alcohols. However, it was already reported that the direct catalytic reduction of acetylene alcohol led to partial racemization [63]. Therefore, to prevent the racemization, acetylene alcohol MαNP ester was subjected to the catalytic reduction, and the diastereomeric purity was checked as follows [13].

**Figure 51:**
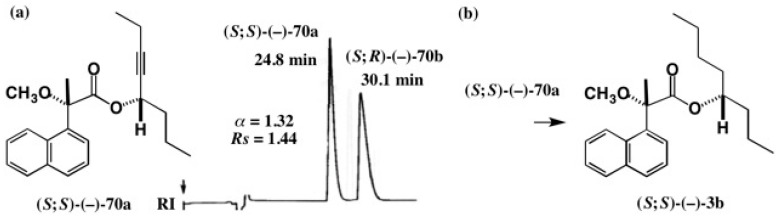
HPLC separation of diastereomeric 5-octyn-4-ol MαNP esters, and conversion to 4-octanol MαNP ester by catalytic reduction: (**a**) **70a**/**70b**, hexane/EtOAc 20:1, reprinted with permission from [60]; (**b**) Reduction with H_2_/PtO_2_ in diethyl ether.

As a model compound, 5-octyn-4-ol was selected, and it was easy to separate its diastereomeric MαNP esters as shown in Figure 51a [60]. The first-eluted MαNP ester (*S*;*S*)-(−)-**70a** was reduced with H_2_/PtO_2_ in diethyl ether yielding 4-octanol MαNP ester (*S*;*S*)-(−)-**3b** [13]: see also Figure 1.

**Figure 52:**
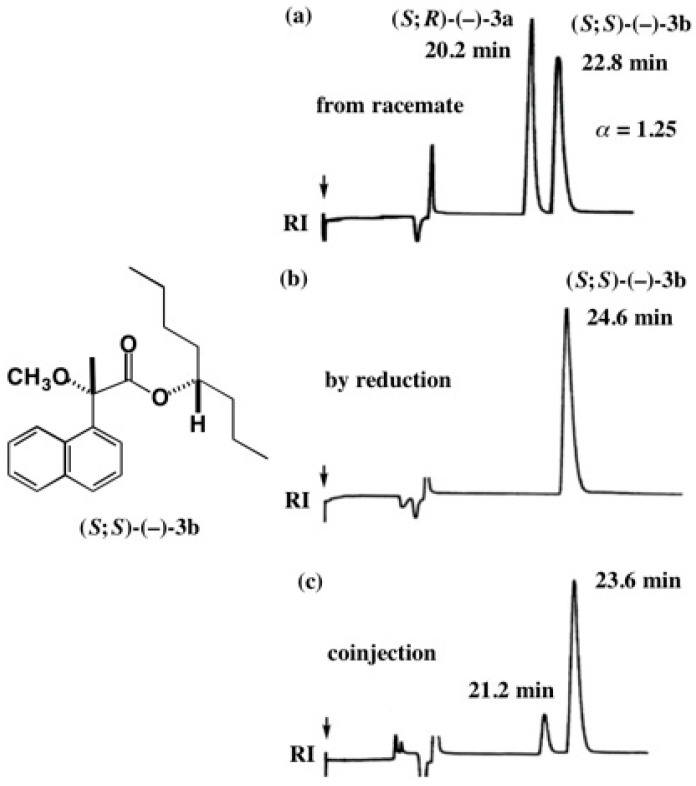
Diastereomeric purity check of MαNP ester (*S*;*S*)-(−)-**3b** obtained by the catalytic reduction of acetylene alcohol MαNP ester (*S*;*S*)-(−)-**70a**. HPLC, hexane/EtOAc 20:1. (**a**) (*S*;*R*)-(−)-**3a** and (*S*;*S*)-(−)-**3b** prepared from racemic 4-octanol (±)-**2**; (**b**) (*S*;*S*)-(−)-**3b** obtained by the reduction of ester (*S*;*S*)-(−)-**70a**; (**c**) coinjection of two samples used in (**a**,**b**). Reprinted with permission from [13].

To check the diastereomeric purity of MαNP ester (*S*;*S*)-(−)-**3b** obtained by the catalytic reduction of acetylene alcohol MαNP ester (*S*;*S*)-(−)-**70a**, HPLC comparison was performed as shown in Figure 52 [13].

Figure 52a shows the HPLC of esters (*S*;*R*)-(−)-**3a** and (*S*;*S*)-(−)-**3b** prepared from racemic 4-octanol (±)-**2**; the part (b) shows the HPLC of ester (*S*;*S*)-(−)-**3b** obtained by reduction of ester (*S*;*S*)-(−)-**70a**; the part (c) shows the coinjection of two samples used in (a) and (b). The HPLC (b) shows only one band indicating that the product was diastereomerically pure, and hence it was concluded that no racemization occurred during the catalytic reduction of the acetylene alcohol MαNP ester [13].

**Figure 53:**
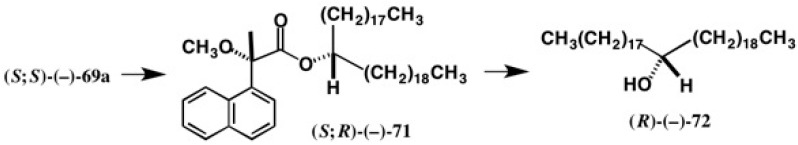
Preparation of enantiopure long chain alcohol with ultimate cryptochirality (*R*)-(−)-**72**.

Next, the ultimate cryptochiral alcohol, (*R*)-(−)-19-octatriacontanol **72**, was synthesized (Figure 53) [13]. Enantiopure acetylene alcohol MαNP ester (*S*;*S*)-(−)-**69a** was subjected to catalytic reduction, yielding saturated alcohol MαNP ester (*S*;*R*)-(−)-**71**. To recover alcohol **72**, a drastic reaction condition was necessary; ester (*S*;*R*)-(−)-**71** was treated with NaOCH_3_ in *iso*-PrOH yielding enantiopure alcohol (*R*)-(−)-**72**: ${{\left[\alpha \right]}_{D}}^{53}$ = −0.038 (σ 0.56, *c* 1.04, CHCl_3_). The specific rotation value was thus very small, and much smaller than the standard deviation σ. Therefore, the observed ${\left[\alpha \right]}_{D}$ value is not reliable, but there is no proper physical data to specify this enantiomer. So, the observed minus sign was used here [13].

**Figure 54:**
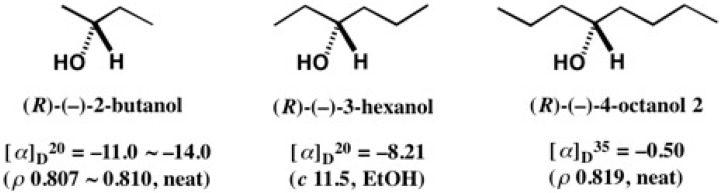
ACs and optical rotation data of alcohol homologs with the structure of CH_3_(CH_2_)_*n*_-CH(OH)-(CH_2_)_*n*+1_CH_3_: (*R*)-(−)-2-butanol [64], (*R*)-(−)-3-hexanol [65], and (*R*)-(−)-4-octanol **2** [13].

The present assignment of the minus sign to alcohol (*R*)-**72** is logically reasonable, when compared with the optical rotation data of (*R*)-(−)-2-butanol, (*R*)-(−)-3-hexanol, and (*R*)-(−)-4-octanol **2** (Figure 54). These are chain alcohol homologs with the structure of CH_3_(CH_2_)_*n_n_*_-CH(OH)-(CH_2_)_*n*+1_CH_3_, where *n* = 0 for 2-butanol; *n* = 1 for 3-hexanol; *n* = 2 for 4-octanol **2**; *n* = 17 for 19-octatriacontanol **72**. Therefore, these compounds should have the same relationship between AC and the optical rotation sign.

**Figure 55:**
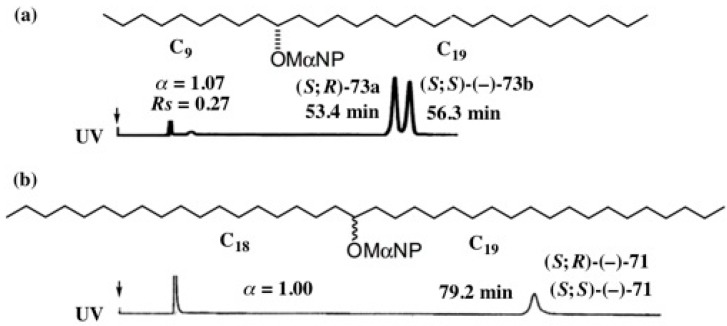
Analytical HPLC of saturated long chain alcohols MαNP esters: silica gel column (10 φ × 300 mm, *n* = 34,400); sample (0.1 mg); detected by UV. (**a**) hexane/EtOAc 80:1; (**b**) hexane/EtOAc 150:1. Redrawn with permission from [13].

It is interesting to study whether the MαNP ester method is still useful for recognizing the chirality of saturated long chain alcohols or not. Figure 55 shows the analytical HPLC of two examples, 10-nonacosanol esters **73a**/**73b** and 19-octatriacontanol esters **71** [13].

In the case of esters **73a**/**73b**, CH_3_(CH_2_)_8_8__- and CH_3_(CH_2_)_18_- groups are compared. It was surprising to find that MαNP esters (*S*;*R*)-**73a** and (*S*;*S*)-**73b** were clearly separated, although two long alkyl chains CH_3_(CH_2_)_8_- and CH_3_(CH_2_)_18_- were compared (Figure 55a). The MαNP acid is thus useful for recognizing the chirality of long alkyl chain alcohols, where two chains are different in length to some extent.

Diastereomeric 19-octatriacontanol MαNP esters **71** were prepared from racemic 19-octa- triacontanol (±)-**72** and (*S*)-(+)-MαNP acid, and the mixture was subjected to analytical HPLC as shown in Figure 55b. However, it was not possible to separate the two esters and they were eluted as a single peak [13]. It may be reasonable, because CH_3_(CH_2_)_17_– and CH_3_(CH_2_)_18_– chains are compared in the case of esters **71**. Namely, the difference is only a –CH_2_ moiety between the CH_3_(CH_2_)_17_– and CH_3_(CH_2_)_18_– groups. This alcohol is thus one of the ultimate cryptochiral compounds, and hence the enantiopure synthesis of such a compound with established AC is extremely difficult.

As shown in Figure 53, however, it was possible to synthesize enantiopure 19-octatriacontanol (*R*)-(−)-**72** with established AC. The MαNP acid method is thus also very powerful for the enantiopure synthesis of such ultimate cryptochiral compounds with established ACs [13].

## 6. Conclusions

We have developed some chiral auxiliaries, such as camphorsultam, CSP acid, CSDP acid, and MαNP acid, which are very useful for the synthesis of enantiopure compounds and simultaneous determination of their ACs. As summarized in Table 1, the prepared diastereomers were separated well by HPLC on silica gel; the separation factor α for CSP esters (average α = 1.09), CSDP esters (average α = 1.20), MαNP esters (average α = 1.58). So, in most cases, HPLC separation on a preparative scale was possible.

Another advantage of the present methods is that the separated derivatives have a high probability to form single crystals suitable for X-ray crystallography. Camphorsultam, CSP acid, and CSDP acid contain heavy atoms such as S and Cl, and hence their ACs could be determined by the X-ray anomalous scattering effect of heavy atoms. In addition, the ACs of these chiral auxiliaries, including MαNP acid, are established and hence they could be used as the internal reference of AC. So, it was easy to determine the ACs from the ORTEP drawings.

It should be noted that the target compound and chiral auxiliary are connected by a covalent bond, not by an ionic bond, and hence the covalently-bonded diastereomers can be separated and purified by HPLC on silica gel. On the other hand, ionic-bonded diastereomers are usually separated by fractional crystallization, and hence in some cases, it was difficult to obtain enantiopure compounds by fractional crystallization.

MαNP acid is very unique, because it can be applied to aliphatic alcohols in addition to aromatic alcohols. Diastereomeric MαNP esters are largely separated by HPLC on silica gel, as shown in Table 1. In this sense, the MαNP acid is superior to the Mosher’s MTPA acid and MPA acid. The ACs of MαNP esters could be determined by ^1^H-NMR diamagnetic anisotropy where ∆δ values are ca. four times larger than those of MTPA esters. In some cases, single crystals of MαNP esters were obtained, and ACs were easily determined from ORTEP drawings. We have never encountered any exception, where the AC determined by ^1^H-NMR disagreed with that by X-ray crystallography. This fact is very important to evaluate the reliability of the ^1^H-NMR diamagnetic anisotropy method using MαNP acid.

The author believes that the diastereomer method using chiral molecular tools explained here would be applicable to a variety of compounds, and hopes that it would be useful for the progress of molecular chirality science.

## Figures and Tables

**Table 1:** Summary of the preparation of diastereomers, HPLC separation, and AC determination by X-ray crystallography and/or ^1^H-NMR diamagnetic anisotropy.

Preparation of Diastereomers	HPLC	AC Determination
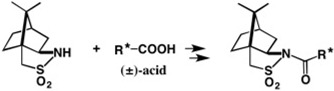	α, not determined *Rs* = 0.7~2.87 *av.* 1.74	X-ray, 6 examples
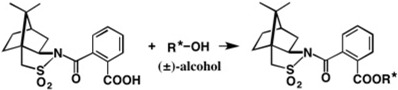	α = 1.05~1.1 *av.* 1.09 *Rs* = 1.3~1.6 *av.* 1.4	X-ray, 3 examples
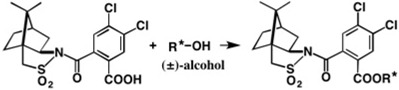	α = 1.14~1.25 *av.* 1.20 *Rs* = 0.91~1.94 *av.* 1.31	X-ray, 4 examples
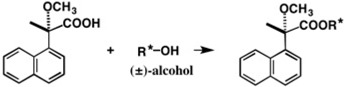	α = 1.15~1.93 *av.* 1.58 *Rs* = 1.18~5.97 *av.* 2.98	^1^H-NMR, 13 examples X-ray, 5 examples

α, separation factor; *Rs*, resolution factor; R*, chiral substituent.
